# DEHP deregulates adipokine levels and impairs fatty acid storage in human SGBS-adipocytes

**DOI:** 10.1038/s41598-018-21800-4

**Published:** 2018-02-22

**Authors:** Kristina Schaedlich, Scarlett Gebauer, Luise Hunger, Laura-Sophie Beier, Holger M. Koch, Martin Wabitsch, Bernd Fischer, Jana Ernst

**Affiliations:** 1 Department of Anatomy and Cell Biology, Martin Luther University, Faculty of Medicine, Grosse Steinstrasse 52, D-06097 Halle (Saale), Germany; 2 Institute for Prevention and Occupational Medicine of the German Social Accident Insurance − Institute of the Ruhr-University Bochum (IPA), Bürkle-de-la-Camp-Platz 1, D- 44789 Bochum, Germany; 3 Department of Pediatrics and Adolescent Medicine, Division of Pediatric Endocrinology and Diabetes Ulm, Eythstrasse 24, D-89075 Ulm, Germany; 4 Present Address: IDT Biologika, Am Pharmapark, D-06861 Dessau-Roßlau, Germany

## Abstract

DEHP is a plasticizer which has been used in plastic products of everyday use for decades. Studies in mice and murine cell culture models identified DEHP as an endocrine disruptor that may also act as an obesogen. As this is of high concern in respect of the worldwide obesity epidemic, our aim is the translation of these findings into a human model system. On the basis of DOHaD, we investigated the influence of an environmentally relevant dose of DEHP [50 µg/ml] on adipogenesis in the human cell culture model SGBS. Pre-adipocytes were exposed to DEHP and differentiated into mature adipocytes. At different stages of differentiation, markers of adipogenesis like GLUT4, FABP4, LPL and PPARs, and of signaling pathways like AMPK/ACC2, JAK/STAT and MAPK were analyzed. Functional markers like adipokine secretion and triglyceride content as well as ROS production were measured in mature adipocytes. We found significantly lower expression levels of adipogenic markers, a reduction in lipid accumulation, higher leptin- and reduced adiponectin levels in the supernatant of treated adipocytes. Moreover, ROS production was significantly elevated after DEHP-exposure. In conclusion, DEHP led to lower grade of adipogenic differentiation in human SGBS-adipocytes under the chosen conditions.

## Introduction

The plasticizer di-(2-ethylhexyl)-phthalate (DEHP) is an additive in plastic products that makes them more flexible and universally deployable. DEHP is not only found in numerous products of daily use, but also in house dust and medical devices like infusion/blood bags and tubing for the administration of blood, plasma and parenteral nutrition. Since February 2015, DEHP is on the REACH (Registration, Evaluation, Authorisation and Restriction of Chemicals) Authorisation List (Annex XIV of REACH) of the European Union (EU), which means that it is subject to an application by producers, importers or users for authorisation for specific uses. Nevertheless, DEHP was the main plasticizer used in all kinds of plastic products for decades and is still ubiquitous^1^. It belongs to the class of endocrine disrupting compounds which act like hormones via specific receptors inducing transcriptional activity. DEHP is lipophilic and leaches (from PVC) into fatty foods, blood and other lipid containing solutions. Exposure to DEHP has been shown to be especially high during medical interventions where medical tubing is employed, such as in (neonatal) intensive care^2–6^. Inoue *et al*. showed that detection levels of DEHP in blood bags range from 1.8 to 83.2 μg/ml, which is in the range of the concentrations tested in this paper^7^. However, human exposure to phthalates varies greatly depending on the population and subgroups (neonates, patients under intensive medical care etc.) and the levels measured are influenced by the analytical methods or specimens (blood, urine, breast milk etc.) that have been used. This issue has been reviewed in more detail by Wittassek and colleagues^8^.

DEHP is one of the high volume industrial chemicals that is classified as an endocrine disruptor but also as an obesogen that promotes adipogenesis^9–12^. Obesogens are chemicals that disrupt normal adipogenesis and fat storage. Some of the known obesogens, e.g. DEHP or its active metabolite mono-(2-ethylhexyl) phthalate (MEHP) respectively, act through the peroxisome proliferator activated receptors (PPARs), the master regulator of adipogenesis^9^.

During the last 2–3 decades, environmental pollution and concomitantly also the prevalence of obesity and diseases of the metabolic syndrome increased tremendously^13^. In the OECD (Organisation for Economic Co-operation and Development) area a majority of the population is overweight (BMI: 25–30) or obese (BMI: 30 ≥ 40).

In Germany 14% of the adult population is obese (OECD data from 2012), and one in five children (5–17 years) is overweight or obese (OECD data from 2010)^14^. Obesity in turn is a major risk factor for diabetes mellitus type II and cardiovascular diseases. A number of studies have shown a link between environmental pollution with industrial chemicals and diseases of civilization^15–18^.

On the base of the Developmental-Origins-of-Health-and-Disease-Hypothesis (DOHaD), our aim was to investigate the influence of an early, time-limited and environmentally relevant exposure to DEHP [50 µg/ml] on development, metabolism and function of differentiating pre-adipocytes in a human *in vitro* model (SGBS). That means, the main question behind this study was, if a time-limited exposure of differentiating SGBS-adipocytes to DEHP (d0-d4), simplifying the situation of the fetus in the womb of the mother, may have an impact on the further differentiation process and with that on the establishment of an obese phenotype in the future. The human Simpson-Golabi-Behmel syndrome (SGBS) pre-adipocyte cell strain is a well characterized *in vitro* model for studies of human adipocyte biology. The cells are neither transformed nor immortalized, and retain their capacity for adipogenic differentiation up to the 50 generations^19^. To our knowledge, this is the first study investigating the impact of DEHP on adipogenesis in a human *in vitro* model. A recent study by Kessler and colleagues on the bioavailability of DEHP has revealed that after oral dosage dose-normalized DEHP blood concentrations over time (area under the curve, AUC) are 50 to 100 times higher in humans compared to rats and marmosets, respectively^20^. In regard to the DEHP metabolite mono-(2-ethylhexyl)-phthalate (MEHP) dose-normalized AUCs in blood were 2 to 8 times higher. Because MEHP is assumed to be the active metabolite of DEHP in endocrine disruption^21^, we also tested if DEHP is hydrolyzed to MEHP in our SGBS culture setting. Furthermore, we were also interested in the influence of DEHP, and its downstream metabolite MEHP, on the oxidative stress level in SGBS cells after 4 days of exposure.

## Results

### DEHP is metabolized to MEHP in SGBS culture

**Figure 1:**
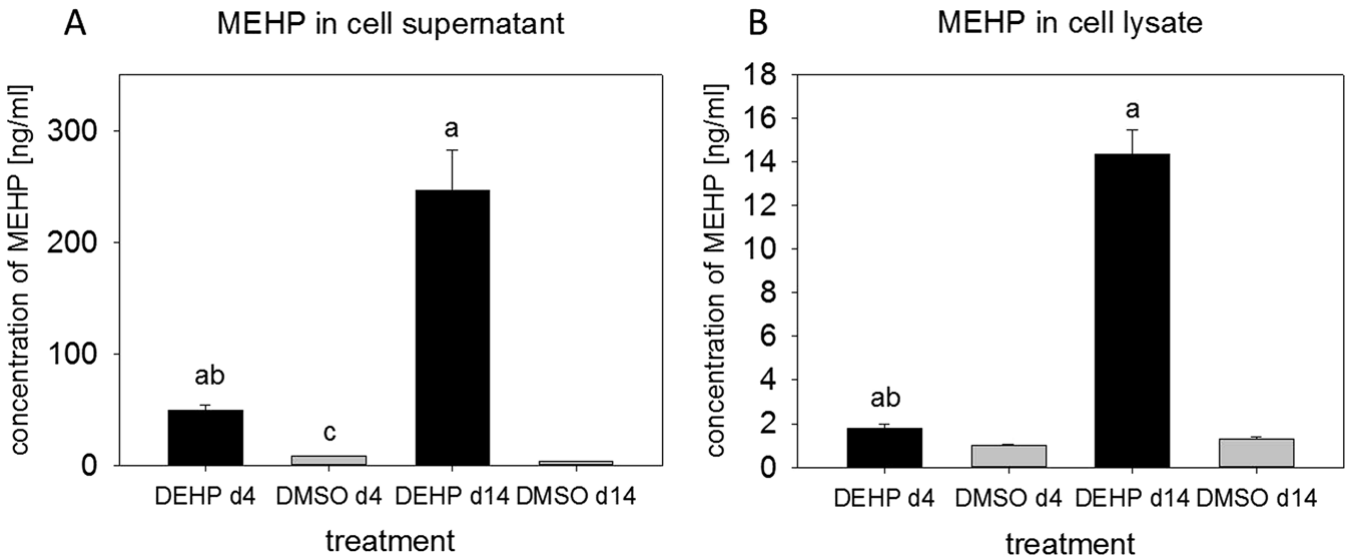
Determination of MEHP in cell supernatants (**A**) and cell lysates (**B**) by HPLC: The SGBS cells were exposed to DEHP [50 µg/ml] for the whole period of adipogenic differentiation (d0–d14). Samples were taken at d4 and d14 for analysis by HPLC. Statistics: a = DEHP versus corresponding DMSO control; b = DEHP d4 versus DEHP d14; c = DMSO d4 versus DMSO d14; ANOVA (Student-Newman-Keuls Method), significance: p > 0.05; N = 4, n = 1 (4 pooled wells each).

MEHP is assumed to be the biologically active metabolite of DEHP^22–24^. For that reason the concentration of MEHP was measured in cell supernatants and cell lysates of SGBS cells exposed to DEHP from d0-d14. MEHP could be detected in both specimen of treated and untreated SGBS cells at d4 and d14 of adipogenesis (Fig. 1). MEHP was significantly increased in all treated samples compared to the corresponding DMSO controls. Furthermore, a significant 4–5 times higher concentration of MEHP was found, comparing DEHP treated supernatants and cell lysates of d4 to d14. In cell supernatants of the DMSO control, a significant reduction of MEHP concentration was found comparing d4 to d14. Nevertheless, in the cell supernatant MEHP concentrations in DMSO controls were very low with 3–8 ng/ml compared to 50–250 ng/ml in DEHP treated samples, and 1–1.3 ng/ml versus 1.7–15 ng/ml in the cell lysate samples. We were also able to detect oxidized metabolites (5OH-MEHP, 5oxo-MEHP and 5cx-MEPP) of DEHP/MEHP in DEHP treated supernatants and cell lysates, but only at concentration levels roughly a factor of 100 lower than MEHP (data not shown).

### DEHP does not affect proliferation of SGBS cells

DEHP has been shown to alter the proliferation rate of different cell types^25–27^. To answer the question if DEHP affects proliferation of the SGBS cells, undifferentiated, proliferating cells were exposed to DEHP for a period of 24 h in a non-confluent stage. After this treatment, there was no significant change in the amount of the proliferating cell nuclear antigen (PCNA) compared to the corresponding DMSO controls (Supplementary Figure S1).

### DEHP leads to an accumulation of reactive oxygen species (ROS)

**Figure 2:**
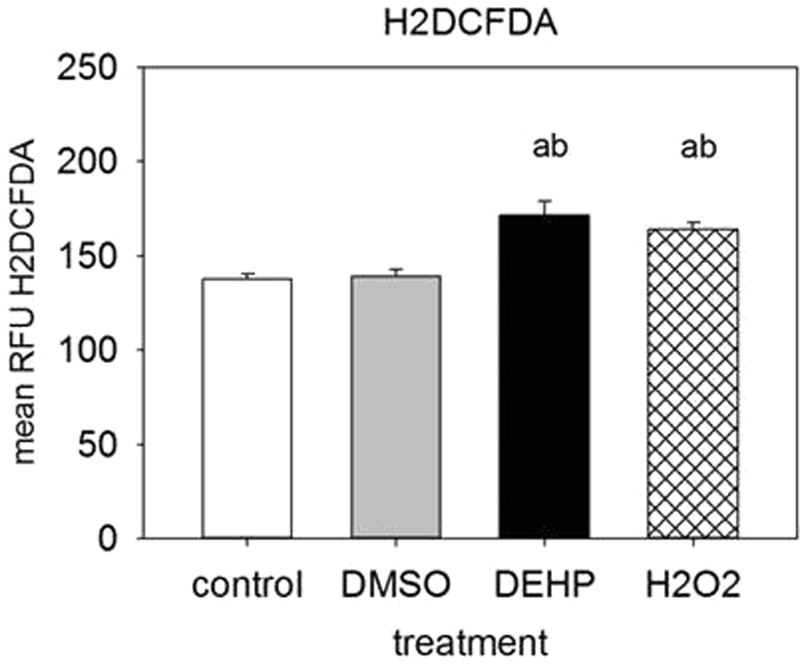
Analysis of ROS-production after DEHP-exposure: SGBS cells were exposed to DEHP from d0–d4 and subsequently differentiated into adipocytes. For the ROS-measurement (A), the medium was changed to DMEM/F12 with 10 µM H2DCFDA at d4 and incubated for 30 min. As the positive assay control H_2_O_2_ (25 µM) was used, as well as an untreated control. Statistics: a = DEHP/H_2_O_2_ versus control; b = DEHP/H_2_O_2_ versus DMSO control; ANOVA (Student-Newman-Keuls Method); N = 2, n = 9.

It has been shown, that DEHP causes oxidative stress in different model systems by elevating ROS levels^28–30^. Though, ROS are not only a stress factor but also crucial for the fate of mesenchymal stem cells (MSC). They are multipotent cells that may differentiate into the osteogenic lineage, neuronal cells or into adipocytes. Among other things, differentiation into adipocytes needs a certain level of ROS, while under lower ROS conditions MSCs are more likely to differentiate into the osteogenic lineage (intensively reviewed by Atashi *et al*.^31^). For those reasons, a H2DCFDA-assay was performed at d4 of adipogenesis, i.e. directly after 4 days of DEHP exposure. H_2_O_2_ and an untreated sample were used as assay control. After DEHP exposure, the ROS level was significantly elevated compared to the corresponding DMSO control and the untreated, but not to the H_2_O_2_ assay control (control: 137.49 ± 2.92; DMSO: 139.30 ± 3,59; DEHP: 171.37 ± 7.52; H_2_0_2_: 164.06 ± 3.76) (Fig. 2A). To further investigate the effects of DEHP on oxidative stress, the ROS-detoxifying superoxide dismutase 2 (SOD2) and the enzyme glutathione peroxidase 1 (GPX1) were analyzed by western blot. The amount of both enzymes was not affected by DEHP at d4 and d8 of adipogenesis (Supplementary Figure S2).

### DEHP decreases the triglyceride content of SGBS-adipocytes

**Figure 3:**
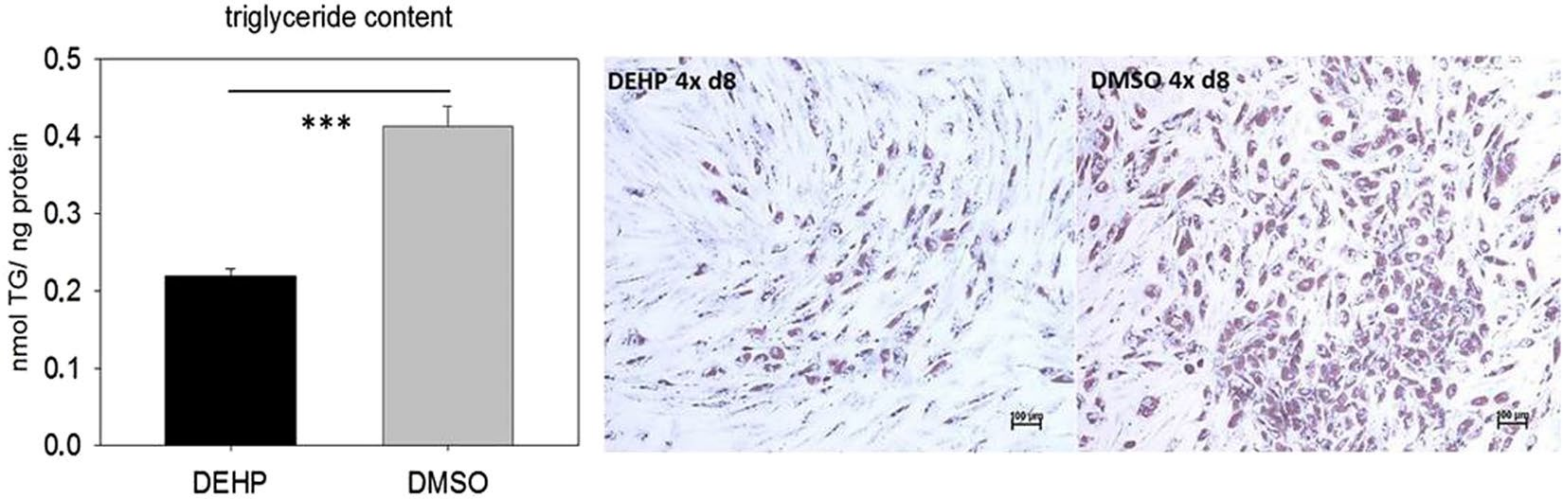
Triglyceride content of adipocytes after DEHP-exposure: SGBS cells were exposed to DEHP from d0–d4 and subsequently differentiated into adipocytes. For the triglyceride measurement (A) the cells were lysed at d8 in a special lysis buffer. Statistics: Student’s t-test (Wilcoxon rank-sum test); N = 2, n = 8. Light microscopy with a 4-fold magnification of Oil Red O stained adipocytes at d8 (B); scale bar = 100 µm.

DEHP is known as an endocrine disruptor with obesogenic effects, mainly investigated in rodent models. To investigate this hypothesis in a human adipogenesis model, SGBS cells were exposed to DEHP in the induction phase from d0-d4. DEHP treatment caused a highly significant reduction of the triglyceride content in SGBS-adipocytes at d8 (DEHP 0.219 ± 0.0096 nmol/ng protein versus DMSO 0.414 ± 0.0246 nmol/ng protein) (Fig. 3A). This effect was also visible after Oil Red O staining. DEHP treated SGBS-adipocytes at d8 showed a lower accumulation of lipid droplets, indicated by less Oil Red O staining compared to the corresponding controls (Fig. 3B).

### DEHP affects adipokine secretion

**Figure 4:**
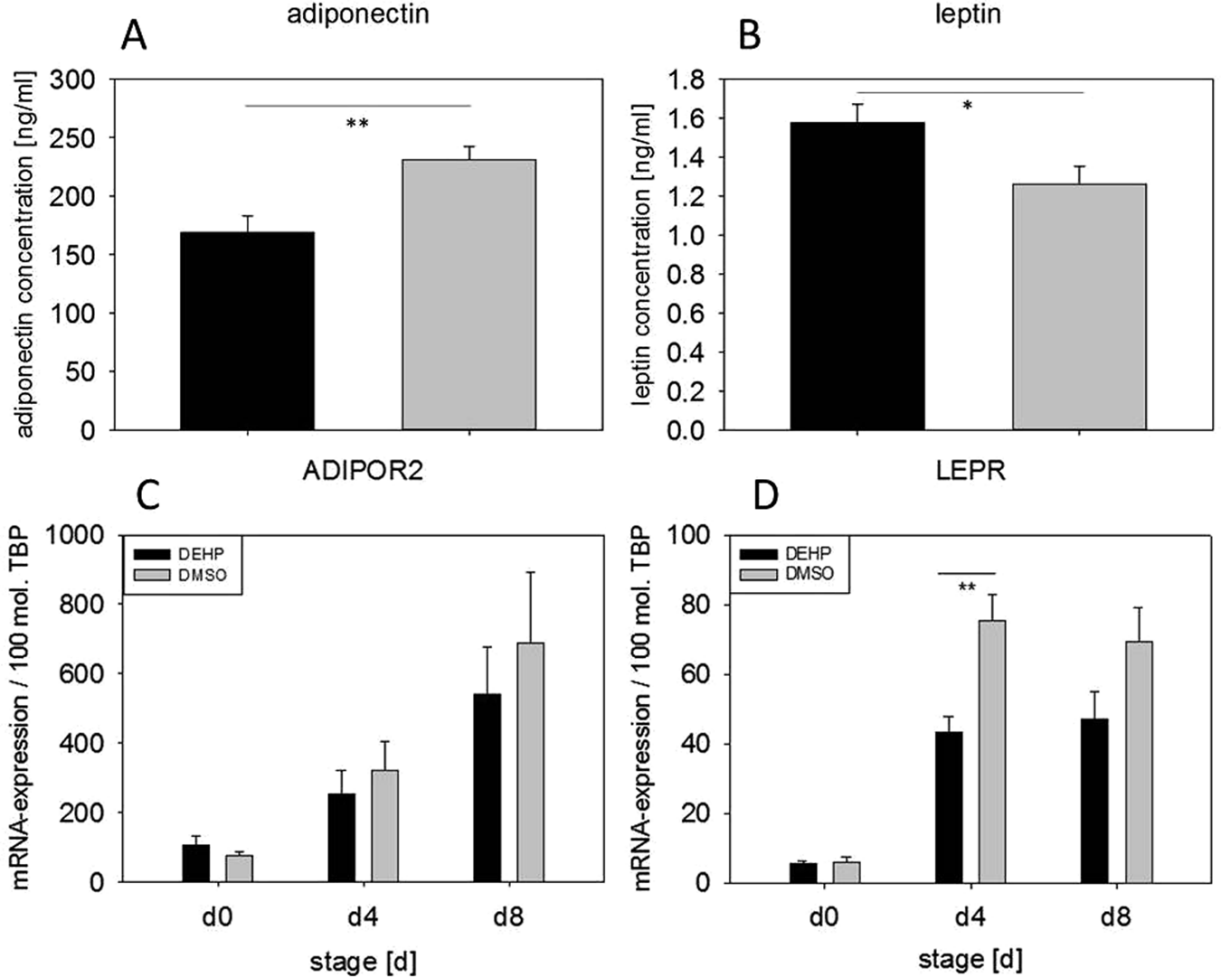
Adiponectin and leptin and their receptors after DEHP-exposure: SGBS cells were exposed to DEHP from d0-d4 and subsequently differentiated into adipocytes. For the adiponectin (**A**) and leptin ELISA (**B**) the cells were analyzed at d8. Statistics: Student’s t-test (Wilcoxon rank-sum test); N = 6, n = 1 (4 pooled wells each). For the analysis of mRNA expression of ADIPOR2 (**C**) and LEPR (**D**) samples were taken at d0, d4 and d8 of differentiation. The housekeeping gene for standardsation was the TATA-box binding protein (TBP). The caption “mRNA expression/mol. TBP” means expression of target gene per molecule(s) TBP. Statistics: Student’s t-test (Wilcoxon rank-sum test); N = 8, n = 1 (4 pooled wells each).

Adipokines like leptin and adiponectin are key markers of adipocyte function and metabolism. Given that DEHP led to a significant reduction in lipid droplets and triglyceride content, the potential mechanisms underlying this observation were further analyzed in SGBS cells exposed to DEHP from d0-d4, during the induction of adipogenesis. At d8 of adipogenesis, a significant decrease in adiponectin secretion (DEHP: 168.84 ± 13.93 ng/ml versus DMSO: 231.21 ± 11.14 ng/ml) was found by ELISA. This decrease in adiponectin levels was accompanied by a significant increase in leptin secretion (DEHP: 1.58 ± 0,09 ng/ml versus DMSO: 1.26 ± 0,09 ng/ml) (Fig. 4A,B). On the basis of these findings, the paracrine effects on the adipocytes were determined by measuring the mRNA expression of the adiponectin receptor 2 (*ADIPOR2*) and the leptin receptor (*LEPR*). The *ADIPOR2* expression showed no changes, while the *LEPR* expression was significantly reduced at d4 (DEHP: 43.33 ± 4.51 mol./100 mol. *TBP* versus DMSO: 75.28 ± 756.17 mol./100 mol. *TBP*), with a trend in reduction at d8 (p = 0.09; DEHP: 47.22 ± 7.69 mol./100 mol. *TBP* versus DMSO: 69.39 ± 9.64 mol./100 mol. *TBP*) (Fig. 4C,D).

### DEHP partially alters adipokine signaling

**Figure 5:**
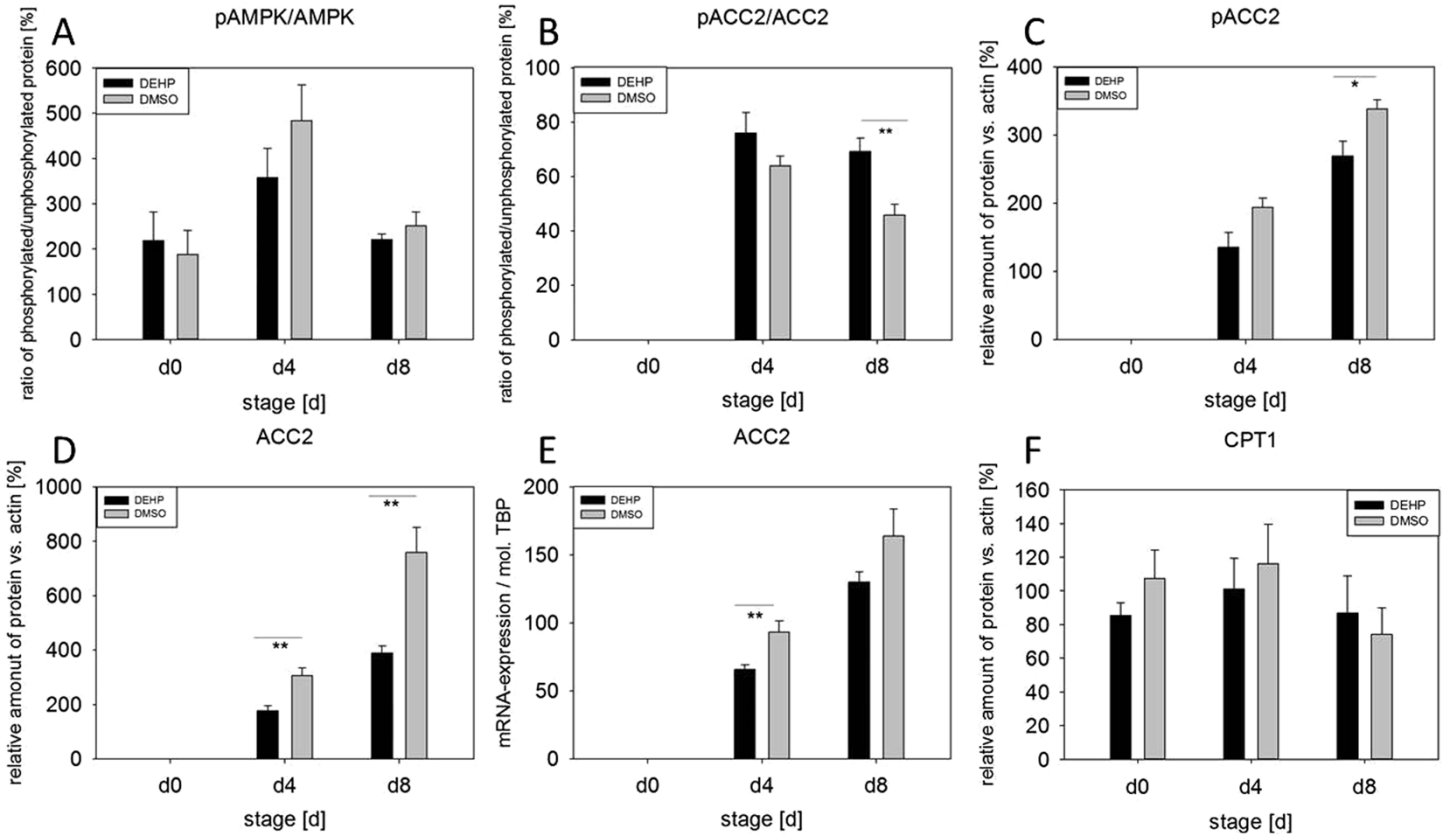
Impact of DEHP on AMPK/ACC2 pathway: SGBS cells were exposed to DEHP from d0-d4 and subsequently differentiated into adipocytes. For western blot analysis of pAMPK/AMPK (**A**), pACC2/ACC2 (**B**), pACC2 (**C**), ACC2 (**D**) and CPT1 (**F**) samples were taken at d0, d4 and d8 of differentiation. Statistics: Student’s t-test (Wilcoxon rank-sum test); N ≥ 4, n = 1 (4 pooled wells each). For the analysis of mRNA expression of ACC2 (**E**) samples were taken at d0, d4 and d8 of differentiation. Statistics: Student’s t-test (Wilcoxon rank-sum test); N = 8, n = 1 (4 pooled wells each).

**Figure 6:**
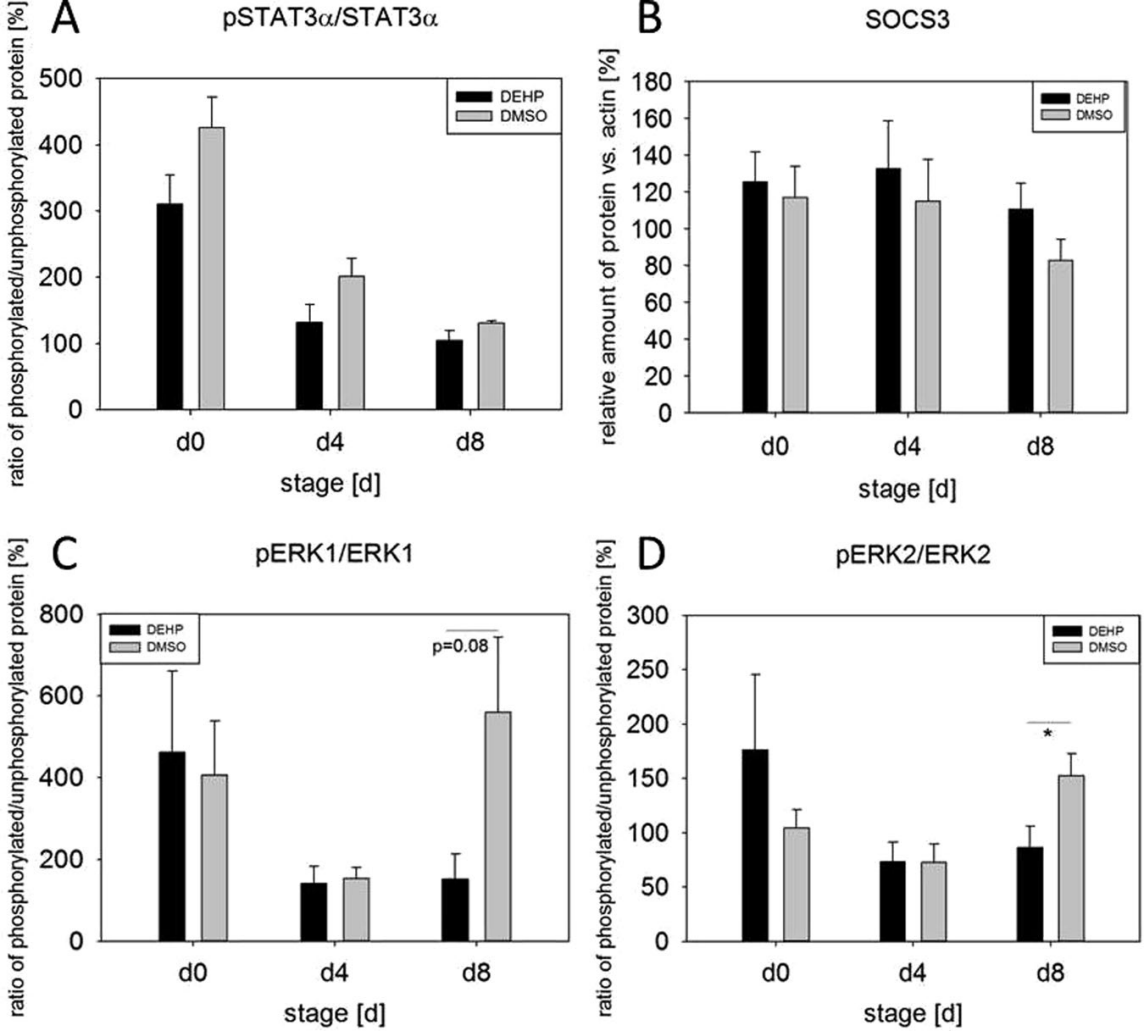
Impact of DEHP on JAK/STAT and ERK1/2 pathway: SGBS cells were exposed to DEHP from d0-d4 and subsequently differentiated into adipocytes. For western blot analysis of pSTAT3α/STAT3α (**A**), SOCS3 (**B**), pERK1/ERK1 (**C**), pERK2/ERK2 (**D**) samples were taken at d0, d4 and d8 of differentiation. Statistics: Student’s t-test (Wilcoxon rank-sum test); N ≥ 4, n = 1 (4 pooled wells each).

The phosphorylation of AMPK (AMP-activated protein kinase), ACC2 (acetyl-CoA carboxylase), ERK1 and ERK2 (extracellular-signal regulated kinases) and STAT3alpha (signal transducer and activator of transcription 3) is presented as a ratio of phosphorylated to non-phosphorylated protein comparing DMSO controls and treated samples. Single proteins like SOCS3 (suppressor of cytokine signaling 3) are expressed as relative amount of protein versus β-actin. The ratio of pAMPK/AMPK was not altered by DEHP-exposure, whereas the ratio of pACC2/ACC2 was significantly increased at d8 (DEHP: 69.27% ± 4.80 versus DMSO: 45.86% ± 3.88) (Fig. 5A,B). If pACC2 is analyzed singly, it was significantly reduced at d8 (DEHP: 269.04% ± 22.28 versus DMSO: 338.13% ± 13.45) (Fig. 5C). Also the amount of ACC2 was significantly reduced a d4 and d8 (d4: DEHP: 176.8 2% ± 18.48 versus DMSO: 306.89% ± 28.09; d8: DEHP: 390.32% ± 25.97 versus DMSO: 760.12% ± 91.26) (Fig. 5D). Furthermore the mRNA expression of *ACC2* showed a significant decrease at d4 (DEHP: 65.76 ± 3.45 mol./mol. *TBP* versus DMSO: 93.32 ± 8.22 mol./mol. *TBP*) after DEHP treatment compared to the corresponding DMSO control (Fig. 5E). However, the amount of the ACC2 downstream target CPT1 (carnitine palmitoyltransferase I) was not altered (Fig. 5F). The analysis of the activation of the JAK/STAT pathway showed no alterations in the ratio of pSTAT3/STAT3, nor in the amount of SOCS3 (Fig. 6A,B). Besides the JAK/STAT pathway, leptin is also known to activate the MAPK (ERK1/2) pathway via its receptor (LepRb)^32,33^. Moreover, both ERK isoforms play distinct but important roles during adipogenesis^34,35^. However, the phosphorylation of ERK2 was significantly reduced at d8 (DEHP: 86.37% ± 19.62 vs. DMSO: 152.27% ± 20.58) with ERK1 showing a strong tendency to a decrease at d8 (p = 0.08), too (DEHP: 151.05% ± 62.54 versus DMSO: 559.92% ± 183.38) (Fig. 6C,D).

### DEHP alters the expression of adipocyte markers

**Figure 7:**
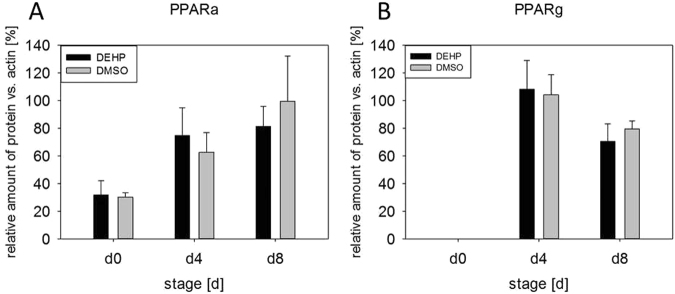
Protein amount of PPARα and PPARγ after DEHP-exposure: SGBS cells were exposed to DEHP from d0-d4 and subsequently differentiated into adipocytes. For western blot analysis of PPARα (**A**) and PPARγ (**B**) samples were taken at d0, d4 and d8 of differentiation. Statistics: Student’s t-test (Wilcoxon rank-sum test); N ≥ 4, n = 1 (4 pooled wells each).

**Figure 8:**
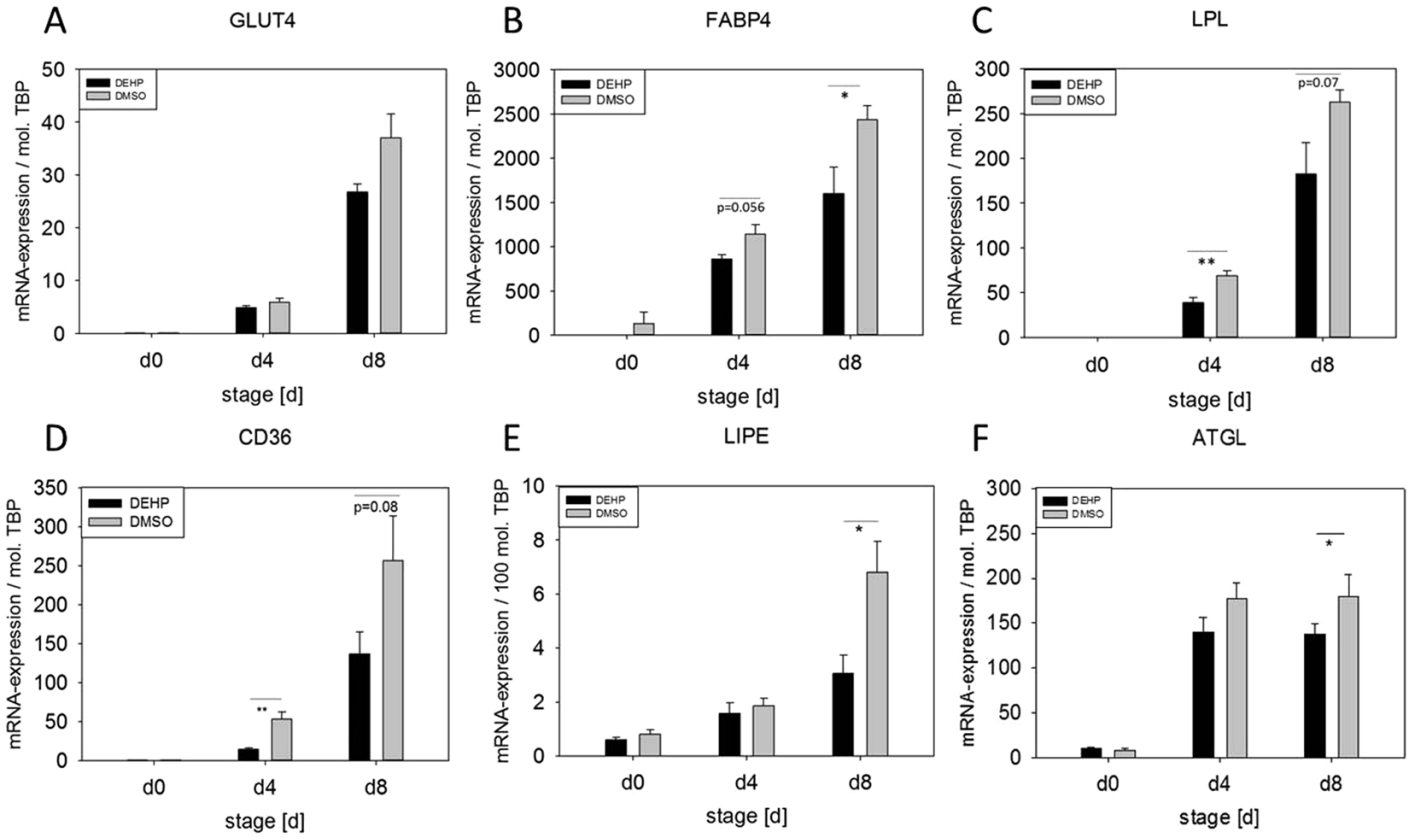
Expression of adipocyte markers after DEHP exposure: SGBS cells were exposed to DEHP from d0–d4 and subsequently differentiated into adipocytes. For the analysis of mRNA expression of FABP4 (**A**), LPL (**B**), GLUT4 (**C**), CD36 (**D**), LIPE (**E**) and ATGL (**F**) samples were taken at d0, d4 and d8 of differentiation; The housekeeping gene for standardization was the TATA-box binding protein (TBP) and the abbreviation “mol. TBP” means molecules TBP. Statistics: Student’s t-test N ≥ 4, n = 1 (4 pooled wells).

**Figure 9:**
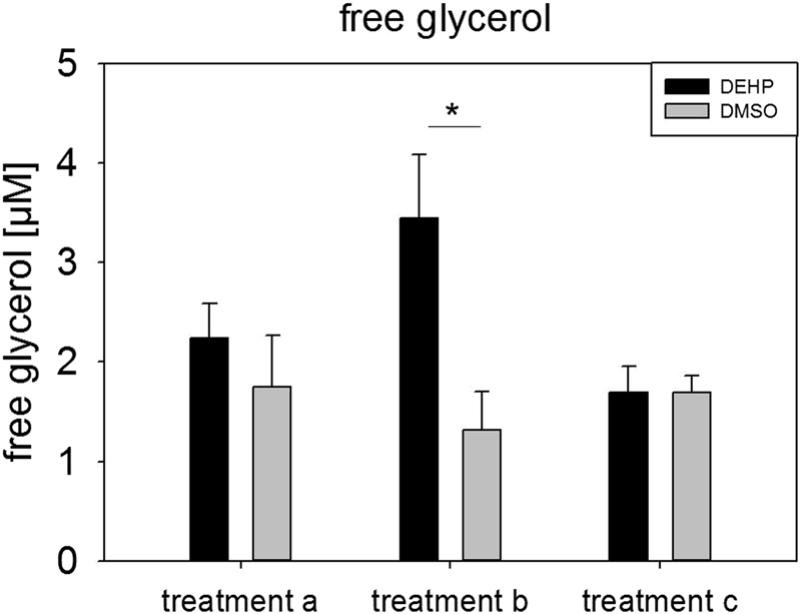
Measurement of free glycerol in SGBS derived adipocytes: The cells have been differentiated as described before and have been treated with DEHP and DMSO as control as followed: treatment a: exposure from d0–d4 and sampling at d4; treatment b: exposure at d4 for 3h and sampling at d4; treatment c: exposure from d0–d4 and sampling at d8. Statistics: Student’s t-test, N = 4, n = 3.

The adipocyte markers PPARa and PPARg (peroxisome proliferator-activated receptors) are crucial transcription factors in adipogenic differentiation and adipocyte function (reviewed by Christodoulides and Vidal-Puig 2010). However, both PPARs were not changed at the protein level (Fig. 7). Also the mRNA expression of *GLUT4* (glucose transporter 4) was unaffected by DEHP treatment (Fig. 8A). Immunohistochemical staining against GLUT4 showed that it was mainly localized in the cytoplasm of the adipocytes of the DMSO controls and also the DEHP treatments (Fig. 9). Nevertheless, the mRNA expression of *CD36* (fatty acid translocase), *FABP4* (fatty acid binding protein 4), *LPL* (lipoprotein lipase) *LIPE* (lipase E, hormone sensitive type) as well as *ATGL (*adipose triglyceride lipase) were significantly decreased after DEHP-exposure if compared to the corresponding DMSO controls (Fig. 8B–F). The mRNA expression of *CD36* was significantly reduced at d4 and tendentially reduced at d8 (p = 0.08) (d4: DEHP: 14.57 ± 2.04 mol./mol. TBP versus DMSO: 53.49 ± 8.7 mol./mol. TBP; d8: DEHP: 136.98 ± 27.86 mol./mol. *TBP* versus DMSO: 256.6 ± 56.93 mol./mol. *TBP)*. The expression of *LIPE* was significantly reduced at d8 (d8: DEHP: 3.06 ± 0.69 mol./100 mol. *TBP* versus DMSO: 6.8 ± 1.15 mol./100 mol. *TBP*) as was *ATGL* (d8: DEHP: 137.9 ± 33.63 mol./mol. *TBP* versus DMSO: 179.94 ± 64.1 mol./mol. *TBP*) and *FABP4* with a trend to decrease at d4 (p = 0.056) (d4: DEHP: 858.66 ± 52.41 mol./mol. *TBP* versus DMSO: 1141.46 ± 107.66 mol./mol. *TBP*; d8: DEHP: 1599.60 ± 299.72 mol./mol. *TBP* versus DMSO: 2435.88 ± 156.40 mol./mol. *TBP*). *LPL* was significantly down-regulated at d4 with a trend to decrease at d8 (p = 0.07) (d4: DEHP: 38.66 ± 5.91 mol./mol. *TBP* versus DMSO: 68.93 ± 5.61 mol./mol. *TBP*; d8: DEHP: 182.28 ± 35.34 mol./mol. *TBP* versus DMSO: 262.52 ± 13.71 mol./mol. *TBP*).

### Direct exposure with DEHP leads to a higher amount of free glycerol

**Figure 10:**
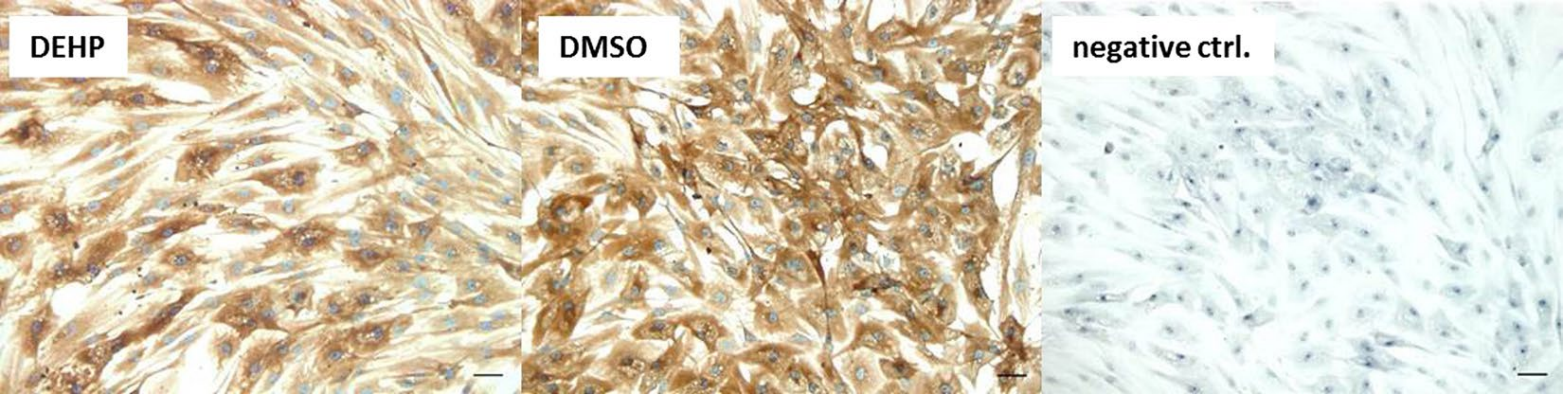
Immunohistochemistry for GLUT4 in SGBS-adipocytes: SGBS cells were exposed to DEHP from d0–d4 and subsequently differentiated into adipocytes. For the analysis of GLUT4 localization within the adipocytes, the cells have been fixed with PFA at d8, stained for GLUT4 (HRP-conjugated antibody – brown staining) and counterstained with hematoxylin (blue staining). The figure shows representative micrographs at × 200 magnification of DEHP treated adipocytes and DMSO controls, as well as the negative control without GLUT4 but secondary antibody. The scale bar indicates 50 µm.

Lipolysis requires three specific enzymes that hydrolyze ester bonds in triglycerides in a three-step process. Those enzymes are ATGL, LIPE, and MGL (monoglyceride lipase). Free fatty acids and free glycerol are the end products of lipolysis and can directly be measured in the supernatant of cells or tissue cultures by commercial assay kits (Cultured Human Adipocyte Lipolysis Assay Kit, Zenbio). To measure the impact of DEHP exposure on lipolysis in SGBS cells, three different treatments have been tested: treatment a: exposure from d0-d4 and sampling at d4; treatment b: exposure at d4 for 3 h and sampling at d4 (as recommended for substance testing in the kit manual); treatment c: exposure from d0-d4 and sampling at d8. The amount of free glycerol was significantly elevated after DEHP exposure in the treatment b, the direct and short-term exposure scenario, whereas the longterm exposure (d0-d4) in treatment a and treatment c did not show any changes (Fig. 10).

## Discussion

On the basis of several studies showing an obesogenic effect of DEHP in murine model systems^36–38^ and in human epidemiological studies^39,40^, we tested this hypothesis in a broader scope in the human SGBS fat cell model. In the present study we demonstrate that the plasticizer DEHP (or its downstream metabolite MEHP) significantly elevated leptin levels and interfered with fatty acid metabolism and lipid storage in a human pre-adipocyte cell model (SGBS). So far, analyses on DEHP-exposure and obesity have mostly been studied in rodent models *in-vivo*^36–38^ and murine cell lines like 3T3-L1 *in-vitro*^41,42^. To date, there are just a few studies that analyzed DEHP/MEHP effects on human adipocyte cell models. For instance, a publication by Campioli and colleagues investigated the impact of MEHP on the relationship between the testicular translocator protein (TSPO) and the PPARs in a human liposarcoma cell line (SW 872). They found an overall induction of adipogenic differentiation after 4 days MEHP-treatment with a significant reduction of PPARg mRNA expression, while it had no effect on triglyceride content. However, this study cannot be compared to our study, because it (i) used a tumor cell line (ii) the cells are cultured in media containing 10% undefined fetal calf serum (iii) the cells are treated with MEHP directly (this is possible due to the FCS used in the media) (iiii) the cells are additionally treated with PMA^43^. Another publication by Ellero-Simatos and colleagues investigated the influence of MEHP on primary cultures of human pre-adipocytes from the subcutaneous adipose tissue fraction. They treated differentiated adipocytes on day 11 with the relatively high concentration of 100 µM MEHP, for 24 and 48 hours, and performed a combined transcriptomic - H NMR metabonomic analysis. The MEHP-treatment led to an activation of the PPAR signaling pathway and other processes involved in lipid metabolism^44^. Although all together excellent studies, the multitude of differences in e.g. cell culture models, culture protocols and study design (among others: primary adipocytes versus SW 872 liposarcoma cells versus SGBS, FCS versus no serum in the media, MEHP- versus DEHP-exposure during different stages of adipogenesis, different duration of exposure) exacerbates a discussion of data among studies. To partly circumvent this fact, the current study design - DEHP-exposure during the induction phase (d0-d4) - was based on data by Biemann and colleagues, who tested 4 different exposure intervals (mixed exposures and single exposures with DEHP, TBT and BPA) in the murine MSC line C3H10T1/2 concerning their impact on different adipocyte endpoints^10,45^. The most effective interval for DEHP-exposure was the induction phase with higher adipocyte numbers and triglyceride levels. As it is known, that mice and men differ especially in regard to the PPAR-family in their ability to be activated by MEHP between isotypes and cell lines^41,42,46^, it is of high importance to transfer murine studies into human models. Using SGBS cells, we could not detect any changes in the amount of PPARa and PPARg. This may be due to several reasons. A likely one are the culture conditions, with rosiglitazone added to the medium between d0 and d4. While on the one hand rosiglitazone is a PPARg agonist and may lead to a maximum in expression and activity and by that masks any possible effect of DEHP, it is known on the other hand that humans have a lower sensitivity towards peroxisome proliferators than rodents^47,48^. Furthermore, we have used a serum-free medium and no fatty acids have been present. Nevertheless, mRNA expression or protein amount does not equal receptor activity. For that reason transactivation assays could reveal actual PPAR activation through DEHP/MEHP in SGBS-adipocytes in future experiments.

Further on, we tested if DEHP-exposure had an influence on proliferation, but could not detect any, which was contradictory to results seen before in different cell types^27,49,50^. Having a closer look at adipocyte morphology, we could show a clearly lower accumulation of lipid droplets by Oil Red O staining and a significant decrease of triglyceride content after DEHP-exposure. This is in high concordance with data from Klöting and colleagues: They exposed mature 3T3-L1 adipocytes to DEHP and found a lower lipid content. Furthermore, they fed obesity-resistant inbred mice with DEHP and found lower circulating adiponectin levels and adiponectin protein in subcutaneous adipose tissue as well as in 3T3-L1 adipocytes^27,49,50^. This was also true in our SGBS-adipocytes at d8 with a significant reduction in adiponectin levels in the cell supernatant of DEHP-treated adipocytes, accompanied by a significant increase of leptin. In principle, a higher leptin level goes together with a higher fat mass, which was the opposite in the present model. But a study by Harris and colleagues supports the present findings by demonstrating, that administration of leptin to leptin-responsive animals reduces body fat mass. They concluded, that leptin inhibits the accumulation of lipids in adipocytes by increasing the turnover of triglycerides, inhibiting basal and insulin-stimulated de novo lipogenesis, but stimulating oxidation of glucose and free fatty acids^51^. Furthermore, Frühbeck and others showed that leptin has an autocrine-paracrine lipolytic effect on isolated murine adipocytes^52–55^. However, leptin is also known to phosphorylate and activate AMPK via its receptor^56^. Activated AMPK in turn phosphorylates and deactivates ACC2, the key enzyme of lipogenesis, which leads to an inhibition of malonyl-CoA formation from acetyl-CoA. In the absence of malonyl-CoA CPT1 is active and supports ß-oxidation^57^. In our study we found a significantly higher ratio of pAAC2/ACC2 after DEHP-exposure, with no changes in AMPK phosphorylation or CPT1 expression. Interestingly, if analyzed singly, the amount of ACC2 and pACC2 was significantly reduced compared to DMSO, as was the mRNA expression. The *LEPR* was significantly down-regulated at d4 with a similar tendency at d8, probably due to a negative feedback mechanism caused by the elevated leptin level. Unfortunately, the paracrine functions of leptin on the adipocyte itself and the LEPR are not well understood. Further on, we analyzed the phosphorylation of STAT3 as a commonly used marker of leptin receptor activation^58^ and SOCS3, a silencer for STAT3, revealing no significant changes in their protein amount. The MAPK (ERK1/2)-pathway is known to be activated by leptin via its receptor and is important for general adipogenesis^32–35^. As Bost and colleagues described different functions of the ERK isoforms in adipogenesis, we analyzed the phosphorylation of ERK1 and ERK2 separately. We found that ERK2 phosphorylation was significantly reduced at d8 with a strong trend of a decreased phosphorylation of ERK1 at d8, too. This result seems not to be very conclusive if one thinks of the elevated leptin levels in the present study. But recent literature on how inhibition of ERK phosphorylation is associated with sevoflurane toxicity in the developing murine brain demonstrated that elevated ROS levels lead to a decreased ERK phosphorylation^59^. As ROS levels were significantly elevated after DEHP-exposure in the present study, this is considered a good reason for the reduction in ERK phosphorylation. However, the mechanisms behind remain unclear and need further investigations. In turn, ERK is responsible for the phosphorylation of Ser660 of LIPE, which together with PKA-activity leads to an activation of LIPE-induced lipolysis^60^. LIPE is a hormone-sensitive lipase that hydrolyzes stored triglycerides from lipid droplets to free fatty acids. The expression of *LIPE* has been shown to be positively correlated with the expression of adiponectin in obese women^61^. Although we measured a significantly lower amount of triglycerides in SGSB adipocytes after DEHP-treatment, we also observed a significantly decreased adiponectin level accompanied by a significant reduction in *LIPE* expression. Quantitatively, *LIPE* and *ATGL* are the most important lipases in the process of lipolysis and by that regulate the catabolism of lipid droplets (intensively reviewed by Zechner *et al*. and Frühbeck *et al*.^62,63^). In the current study the expression of both enzymes of lipolysis (*LIPE* and *ATGL*) was downregulated at d8. Nevertheless, there was a significantly lowered amount of triglycerides after DEHP-exposure in SGBS-adipocytes of d8. Therefore, a direct measurement of lipolysis was conducted to improve the interpretation of these results, as mRNA expression does not necessarily reflect the activity of an enzyme. Yet, studies in mice observed a correlation between mRNA expression of *LIPE* and *ATGL* with the grade of lipolysis^62^, while others showed that in fasted human white adipose tissue (WAT) ATGL expression is reduced with increased ATGL protein concentration and increased lipase activity. According to Schweiger and co-workers, the reason for that is posttranscriptional regulation^64^. This correlates with our observation of a significantly elevated amount of free glycerol 3 hours after a direct exposure to DEHP, significantly reduced expression levels of *ATGL* and *LIPE* and a reduced triglyceride accumulation in lipid droplets. The finding that no changes were seen in free glycerol levels in treatment a and c (DEHP exposure: d0-d4) may be probably due to the fact, that lipolysis has taken place at the beginning of exposure and was finished due to a lack of TGs and changed metabolic conditions at the time point of measurement at d4 and d8. This needs to be further validated in future studies. As a reduced amount of triglycerides could also be due to a reduced glucose or fatty acid uptake, we also investigated the expression of *FABP4*, *CD36*, *LPL*, and *GLUT4*. The cytoplasmic protein FABP4 binds long-chain fatty acids and other hydrophobic ligands and is involved in fatty acid uptake, transport, and metabolism. Interestingly, *FABP4* bound to fatty acids physically interacts with phosphorylated LIPE and by that can form a FABP4:pLIPE complex at the lipid droplet to facilitate LIPE-dependent lipolysis^63,65^. In our studies, both, *LIPE* and *FABP4*, were significantly down-regulated after DEHP-exposure. Moreover, our results on *FABP4* and also *leptin* expression are in concordance with a study by Gan and colleagues who found that *FABP4* was down-regulated by leptin assuming FABP4 and leptin to play opposite roles in the regulation of fatty acid oxidation^66^. The fatty acid translocase CD36 is another key player in adipose function that also interacts with FABP4^67–69^. According to Coburn and others, CD36 deficiency is associated with an impaired fatty acid uptake and an increased basal lipolysis^63,70^. In our studies the expression of the fatty acid translocase *CD36 and FABP4* was significantly decreased after DEHP exposure, pointing towards less fatty acid uptake and transport. This is further supported by the significantly down-regulated expression of *LPL* in our study. *LPL* functions as triglyceride hydrolase and is responsible for receptor-mediated lipoprotein uptake. In studies with ob/ob mice leptin decreased LPL-activity^71^ which is in accordance with our findings. GLUT4 is a protein that functions as an insulin-regulated facilitative glucose transporter. Within minutes of insulin stimulation, the protein translocates to the cell surface and transports glucose across the cell membrane. DEHP did not change the mRNA expression of *GLUT4*, nor did it alter the localization of *GLUT4* if compared to the control.

A likely candidate for DEHP-interference with adipogenesis are reactive oxygen species (ROS). It has been shown before that DEHP causes oxidative stress^25,28,29^ and that it plays a role in adipogenic differentiation^72,73^. Moreover, ROS are associated with a decreased secretion of adiponectin in 3T3-L1 cells^74^ and also in human studies^75^, which is in line with our findings. Furthermore, as mentioned above, ROS seem to lead to a decreased ERK phosphorylation^59^ and thus may influence adipogenesis in general. In the present study, the H2DCFDA-assay revealed a significant elevation of the ROS level at d4 in DEHP-treated SGBS cells and a significant decrease in adiponectin in the cell supernatant. However, no changes could be observed in the protein amounts of the ROS-detoxifying enzymes *GPX1* and *SOD2*. Wang and colleagues had similar results, when they investigated the influence of DEHP [1, 10 and 100 µg/ml] on cultured antral follicles. Although they measured a significant elevation in ROS levels, they could not detect any changes in *GPX1* and *CAT* expression, but a decrease in *SOD1* expression in the 10 µg/ml dose group. However, the question why in the present study no changes could be observed at least in the amount of SOD2 remains unanswered.

## Conclusion

We could not confirm an obesogenic effect of DEHP in SGBS-adipocytes under the chosen conditions. In contrast, our results clearly indicate that DEHP-exposure during the induction phase of adipogenesis led to a significantly lower degree of adipogenic differentiation, a higher lipolytic activity and a reduced uptake and transport of fatty acids into and within the adipocytes. Besides this, it is likely that ROS play a more important role in the effects of DEHP than so far expected. Furthermore, we showed that there are species differences between human and murine models that must be considered when translating murine data into human context.

## Material and Methods

### Culture conditions and DEHP-exposure

The SGBS pre-adipocyte cell strain was obtained from the laboratory of Prof. Martin Wabitsch *et al*.^76^. SGBS pre-adipocytes were grown to near confluence and then incubated (d0) in a serum-free differentiation medium [2 μmol/l rosiglitazone (Cayman #714740), 25 nmol/l dexamethasone (Sigma Aldrich #D-1756), 0.5 mmol/l methylisobuthylxantine (Sigma Aldrich #I-5879), 0.1 μmol/l cortisol (Sigma Aldrich #H-0888), 0.01 mg/ml transferrin (Sigma Aldrich #T-2252), 0.2 nmol/l triiodothyronine (Sigma Aldrich #T-6397), and 20 nmol/l human insulin (Sigma Aldrich #19278)]. After 4 days the medium was changed, and the cells were further cultured in medium supplemented with 0.1 μmol/l cortisol, 0.01 mg/ml transferrin, 0.2 nmol/l triiodothyronine, and 20 nmol/l human insulin. The SGBS pre-adipocytes were exposed to DEHP [50 µg/ml] (Sigma Aldrich) from d0-d4. DEHP had been solved in DMSO (Sigma Aldrich) in a 1000-fold stock solution. The maximum concentration of DMSO in the culture media was 0.1%. Controls were run as vehicle controls with 0.1% DMSO in the media. The dose used is comparable to DEHP doses found in neonates undergoing clinical procedures, such as transfusion or extracorporeal membrane oxygenation as well as in whole blood and blood components^77–79^. All experiments have been finalized at d8 of differentiation.

### HPLC analysis of MEHP in supernatants and cell lysates

MEHP is negatively charged and by that cannot be taken up into the cells in an *in vitro* model cultured without fetal calf serum (FCS)^80,81^. Moreover, the systemic availability of DEHP is 50–100 times higher in humans than in marmosets and rodents^20^. Therefore we used DEHP as the primary agent and tested whether DEHP is metabolized to the active metabolite MEHP in SGBS cells. Only in the case of the HPLC-analysis of DEHP-metabolisation to MEHP, SGBS cells were cultured until d14 and exposed to DEHP from d0 to d14 of adipogenic differentiation. The data obtained from this part of the study was basic for the whole study design, as it aimed to reveal DEHP-degradation in the SGBS cell model in general and the grade of accumulation of e.g. MEHP in the cells and in the media over time. Due to that, samples of media and cell lysates were obtained at d4 and d14. The assays used to determine the DEHP metabolite MEHP (and other oxidized DEHP metabolites) were performed in the medium and cell lysates using high-performance liquid chromatography tandem mass spectrometry with quantification via isotope dilution (Koch *et al*., 2003). The selectivity and specificity of such an HPLC-MS/MS method (including isotope labelled internal standards) can be considered as a gold standard for a robust, sensitive and specific determination of such biomarkers. The respective LOQs for MEHP (and other phthalate metabolites) are 0.5 µg/l for MEHP and 0.2 µg/l for the oxidized metabolites. The extensive chromatographic separation and the specific mass transitions ensure that “cross-reactivity” (in LC-MS/MS better termed interference) with or by other phthalates or phthalate metabolites can be excluded. Sample measurements were carried out by the IPA laboratory in Bochum (Germany), which has successfully participated as a reference laboratory for phthalate metabolite analyses in the quality assurance programme of the European Union financed Consortium to Perform Human Biomonitoring on a European Scale (COPHES)^82^.

### Analysis of cell proliferation by Proliferating-Cell-Nuclear-Antigen (PCNA) western blot

SGBS cells were seeded on 12-well plates and cultured in basal medium^19^ for 2 days. Afterwards the medium was changed, now containing 50 µg/ml DEHP for 24 h. The exposed SGBS cells were harvested with RIPA buffer [10% 10 X PBS, Nonidet NP40, 10% SDS, 0.5% sodiumdeoxycholate, protease inhibitors, phosphatase inhibitors (Roche, Germany)] and the protein was used for western blot analysis with the PCNA antibody^83^.

### Quantitative real time PCR (qRT-PCR)

Total RNA of SGBS cells has been purified with the RNeasy Lipid Tissue Mini Kit (Qiagen) and was quantified by UV/VIS-spectrometry at 260 nm using the NanoVue system (GE Healthcare). The reverse transcription reaction was performed with 1 µg of total RNA using the RevertAid^™^ H Minus Reverse Transcriptase (Fermentas, Germany), following the instructions of the Fermentas manual. The samples were analyzed by qRT-PCR using an iQ5 Optical System (Bio-Rad Laboratories, Herts, UK) and SYBR green master mix with fluorescein (Eurogentec, Germany) as an intercalating fluorescent dye specific for double stranded DNA. Primer sequences are listed in Table 1. All primers used were tested for specificity by sequencing of the PCR products. Absolute mRNA expression was calculated by target specific plasmid standards using serial dilutions. Each assay was run with duplicates of each cDNA sample as well as a no template control (NTC) and the target specific standard which also served as a positive control. The expression of the housekeeping gene TATA-box binding protein (TBP) was used to normalize samples for the amount of cDNA used per reaction. To confirm the amplification of only one single target, the resulting qRT-PCR products were analyzed by a melting curve. If possible, exon-spanning primers have been used.

**Table 1:** Primer sequences.

*target*	primer	sequence	amplicon [bp]
*TBP*	*forward*	TGTGCTCACCCACCAACAAT	199
	*reverse*	AGTCGTCTTCCTGAATCCCT	
*ADIPOR2*	*forward*	GAGACACGCGGATCAACTCA	175
	*reverse*	GTTGGTGCCCTTTTCTGAGC	
*LEPR*	*forward*	ACACCAGAGTGATGCAGGTTT	187
	*reverse*	ATGCTCAAACGTTTCTGGCTTC	
*ACC2*	*forward*	ACAGTCCTGAGATCCCCCTC	238
	*reverse*	GTTCAGCCGGGTGGACTTTA	
*GLUT4*	*forward*	ACTGGCCATTGTTATCGGCA	213
	*reverse*	GTCAGGCGCTTCAGACTCTT	
*LPL*	*forward*	AGTCCCGGCTTCGCCATTCA	172
	*reverse*	TCGCCCAGTTTCAGCCTGACT	
*FABP4*	*forward*	GGGTCACAGCACCCTCCTGAAA	162
	*reverse*	TGGTGGCAAAGCCCACTCCTACT	
*CD36*	*forward*	ACTGAGGACTGCAGTGTAGGA	216
	*reverse*	ACAAGCTCTGGTTCTTATTCACA	
*LIPE*	*forward*	GTGCTAGGCACATAGCCTCC	166
	*reverse*	TGATGGCACTTCCTCTTGGG	
*ATGL*	*forward*	TATCCCACTTCAACTCCAAG	156
	*reverse*	GTGTTCTTAAGCTCATAGAGTG	

### Western blot analysis

For immunodetection, the SGBS cells were harvested and lysed in RIPA buffer [10% 10 x PBS, Nonidet NP40, 10% SDS, 0.5% sodiumdeoxycholate, protease inhibitors, phosphatase inhibitors (Roche, Mannheim, Germany)]. Total protein lysates were separated by SDS-PAGE and electrotransferred to nitrocellulose membranes. Blots were blocked in 0.1% TBST with 3% (wt/vol) BSA for 2 hours. The primary antibody was incubated at 4 °C overnight. Antibodies were used as follows: pAMPK (1:500, mAB#2535, Cell Signaling, Boston, USA), AMPK (1:500, #2532, Cell Signaling), pACC2 (1:500, GTX62736, Gene Tex), ACC2 (1:500, GTX89089, Gene Tex), ß-actin (1:10000, A5441, Sigma Aldrich), GPX1 (1:500, HPA044758, Sigma Aldrich), SOD2 (1:500, HPA001814, Sigma Aldrich), ERK (1:500, #9170 Cell Signaling), pERK (1:1000, #4370, Cell Signaling), STAT3 (1:500, #4904, Cell Signaling), pSTAT3 (1:1000, mAb#9145, Cell Signaling), SOCS3 (1:250, #2923, Cell signaling), PPARa (1:500, 101710-500, Cayman), PPARg (1:250, mAb2443S, Cell signaling) and PCNA (1:1000, mAB#13110, Cell Signaling). The secondary antibodies (horseradish peroxidase-conjugated) were goat anti-mouse (1:20000, Dianova) or goat anti-rabbit (1:6000, DAKO), respectively. The protein amount was calculated as the ratio of each protein versus ß-actin intensity. Western Blot images are shown in Supplementary Fig. S3.

### Enzyme-linked immunosorbent assay (ELISA)

The Quantikine^®^ ELISA Human Total Adiponectin/Acrp30 (BioVendor, Germany) was used to determine the concentration of adiponectin in adipocyte supernatants at day 8. The Leptin ELISA (high sensitive) (IBL international, Germany) was used to determine the concentration of leptin in adipocyte supernatants at day 8. Both kits were handled according to the manufacturer’s instructions.

### Triglyceride Assay

The Adipogenesis Kit (Sigma Aldrich) was used to detect total cellular concentrations of triglycerides by a coupled enzyme assay, which results in a colorimetric product, proportional to the trigylcerides present at day 8. The procedure followed the manufacturer’s instructions.

### Oil Red O staining of adipocytes

First the adipocytes were gently rinsed with sterile PBS. Afterwards 2 ml of 10% paraformaldehyde were added to the cells followed by an incubation of 1 hour at room temperature. All procedures involving formalin were performed in a fume hood. The stock solution of Oil Red O was prepared with 300 mg of Oil Red O powder added to 100 ml of 99% isopropanol. The working solution consisted of 3 parts of Oil Red O tock solution and 2 parts deionized water. It had to sit at room temperature for 10 minutes and was stable for no longer than 2 hours. Afterwards the working solution was filtered completely through a filter funnel. For the Oil Red O staining, the formalin was removed from the cells followed by a washing step with sterile water. After removal of the water, the cells were incubated with 2 ml of 60% isopropanol for 5 min. For the Oil Red O staining the isopropanol was removed and 2 ml of the working solution were added to the adipocytes for 5 min. The Oil Red O was indirectly rinsed with tap water until the water ran clear. To counterstain the cells 2 ml of hematoxylin were added and incubated for 1 min. The adipocytes were rinsed again with warm tap water until the water ran clear. The plates were kept wet with water until light microscopy analysis.

### Measurement of free glycerol in cell supernatants

The measurement of free glycerol was conducted in supernatants of living SGBS cells in three different treatments. For treatment a and c the SGBS cells have been exposed to DEHP [50 µg/ml] or DMSO (control) during the induction phase from d0-d4. For treatment a, the sampling took place at d4 and for treatment c at d8. Treatment b was different from the usual exposure window of this study as exposure took place at d4 for 3 hours right before measurement. This was in accordance with the kit’s manual for substance testing. The whole measurement procedure was conducted as described in the manual (Cultured Human Adipocyte Lipolysis Assay Kit, Zenbio).

### H_2_DCFDA-Assay for detection of reactive oxygen species (ROS)

For the analysis of ROS, SGBS cells have been seeded into black 24-well plates and exposed and differentiated into adipocytes as described before (2,1.). At d4 and d11 the medium was changed to DMEM/F12 (Gibco® # 31330-038) with 10 µM H_2_DCFDA (Thermo Fisher Scientific), dissolved in DMSO and incubated for 30 min at 37 °C in the absence of light. Afterwards the cells have been washed with medium. Measurement was carried out with fresh media in a plate reader at Ex/Em: 495 nm/535 nm. The dye is oxidized in the cells and through that gives a detectable fluorescence signal. As the positive assay control H_2_O_2_ (25 µM) was used as well as an untreated control.

### Immunohistochemistry

The glucose transporter 4 is a transporter that translocates to the membrane, if activated by insulin. Without insulin stimulation, GLUT4 is inactive and located in the cytoplasm of the adipocyte. By immunohistochemical staining we aimed to analyze the localization of GLUT4 after DEHP-exposure. For the culture of the SGBS cells, 4-well chamber slides (Sarstedt) were used. The cells were seeded in a density of 0.5 × 10^4^ per well. DEHP-exposure and differentiation were as described before (2). At d8 the SGBS-adipocytes were fixed with 4% paraformaldehyde for 25 min followed by three washing steps with PBS and one washing step with PBS + Tween 20 [0.1%] (PBST) for 5 min. Afterwards the endogenous peroxidases were inactivated with 3% H_2_O_2_ in methanol for 20 min at room temperature in the absence of light. This was again followed by washing steps with H2O (2x , 5 min) and PBST (2x , 10 min). Afterwards the adipocytes were blocked with 10% goat serum for 60 min at room temperature. Next, the primary antibody (GLUT4, 1:300, 4048, Sigma Aldrich) in 3% (wt/vol) bovine serum albumin (BSA)/PBS was added and incubated overnight at 4 °C. The cells were washed three times with PBST and incubated with the secondary antibody Dako EnVision + System-HRP labeled Polymere anti mouse (1:1 in PBS). Diaminobenzidine (DAB; WAK-Chemie Medical) was used for detection. The DAB reaction was stopped in water after 10 min. The cells were counterstained with hematoxylin and subsequently embedded on Superfrost slides (Menzel Gläser) using 4.8 g of Mowiol reagent (Calbiochem) dissolved in 12.0 g of glycerol (Merck). All steps were performed within the same experiment, examined microscopically during the same session, using identical microscope and camera settings (BZ 8100, Keyence).

### Statistical Analyses

All data obtained from qRT-PCR and western blotting were run in at least four independent biological experiments (N ≥ 4), and presented as mean ± standard error of the mean (SEM). Four wells have been pooled within one biological experiment (N = 1, n = 1 (4 pooled wells)) to obtain sufficient material for mRNA or protein extraction. For ROS-detection and the adipogenesis assay the independent biological experiments were run two times (N = 2) with at least 6 individual replicates (n = 6) per experiment (N). Experiments for the adipokine ELISAs were run in 6 independent biological replicates (N = 6) with 1 individual replicate (n = 1). In the case of qRT-PCR, ELISA, H2DCFDA- and adipogenesis assay the samples have been measured in two technical replicates and the mean has been calculated and used for statistical analyses. For statistical analysis Student’s t-test or, if necessary, the Wilcoxon rank-sum test was performed. In the case of multiple comparison procedures, the one-way analysis of variance (ANOVA) or, if necessary, the Student-Newman-Keuls Method was performed. The tests were done using Sigma Plot software and are mentioned in figure legends, too.

The datasets generated during and/or analysed in the current study are available from the corresponding author on reasonable request.

## Electronic supplementary material

Supplementary Information
