# Supplementary material for: DEHP deregulates adipokine levels and impairs fatty acid storage in human SGBS-adipocytes
Source: Sci Rep. 2018 Feb 22;8:3447. doi: 10.1038/s41598-018-21800-4 (PMC5823900; doi:10.1038/s41598-018-21800-4)
Supplement: Supplementary file 1 — Supplementary Information [file 41598_2018_21800_MOESM1_ESM.docx]

***Supplementary Information***

**DEHP deregulates adipokine levels and impairs fatty acid storage in human SGBS-adipocytes**

*Kristina Schaedlich* ^a^, Scarlett Gebauer ^a1^, Luise Hunger ^a^, Laura-Sophie Beier ^a^, Holger M. Koch ^b^, Martin Wabitsch ^c^, Bernd Fischer ^a^ and Jana Ernst ^a^*

^a^ Department of Anatomy and Cell Biology, Martin Luther University, Faculty of Medicine, Grosse Steinstrasse 52, D-06097 Halle (Saale), Germany;

E-mail: kristina.schaedlich@medizin.uni-halle.de, luise.hunger@student.uni-halle.de, laura-sophie.beier@student.uni-halle.de, bernd.fischer@medizin.uni-halle.de, jana.ernst@medizin.uni-halle.de

^1^ IDT Biologika, Am Pharmapark, D-06861 Dessau-Roßlau, Germany; E-mail: scarlett.gebauer@gmx.de

^b^ Institute for Prevention and Occupational Medicine of the German Social Accident Insurance − Institute of the Ruhr-University Bochum (IPA), Bürkle-de-la-Camp-Platz 1, D- 44789 Bochum, Germany; E-Mail: koch@ipa-dguv.de

^c^ Division of Pediatric Endocrinology and Diabetes Ulm, Department of Pediatrics and Adolescent Medicine, Eythstrasse 24, D-89075 Ulm, Germany; E-mail: Martin.Wabitsch@uniklinik-ulm.de

**Supplementary Figure S1:**


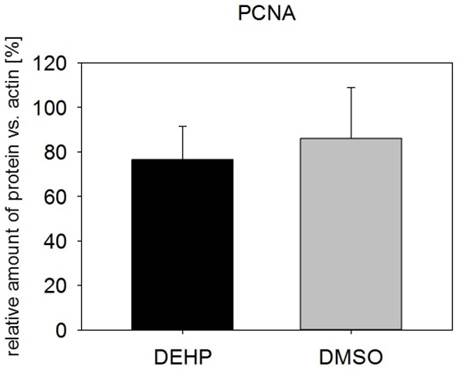


**Supplementary Figure S1:** Determination of proliferation after DEHP-exposure. SGBS cells were exposed to DEHP for 24 h in a non-confluent state. Afterwards the SGBS cells were harvested and the protein was used for western Blot analysis with an antibody against PCNA. Statistics: Student’s t-test (Wilcoxon rank-sum test); N=4, n=1 (4 pooled wells each).

**Supplementary Figure S2:**

**
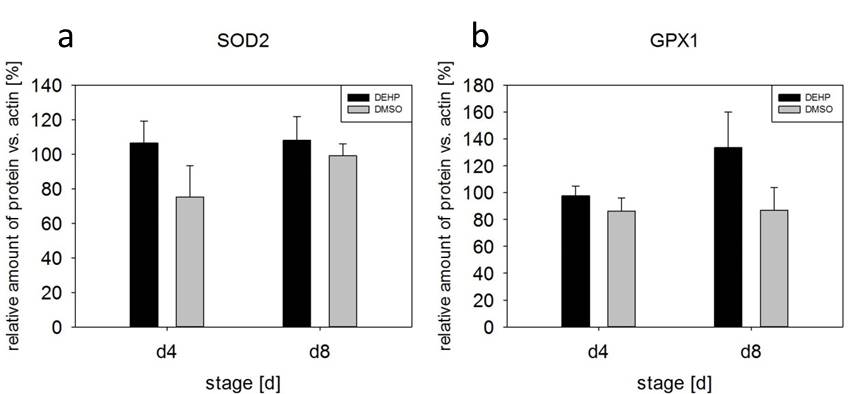
**

**Supplementary Figure S2:** Analyses of ROS associated enzymes after DEHP-exposure. SGBS cells were exposed to DEHP from d0-d4 and subsequently differentiated into adipocytes. For the Western blot analysis of SOD2 (A) and GPX1 (B) protein samples were obtained at d4 and d8; Statistics: Student’s t-test (Wilcoxon rank-sum test); N=4, n=1 (4 pooled wells each).

**Supplementary Figure S3:**


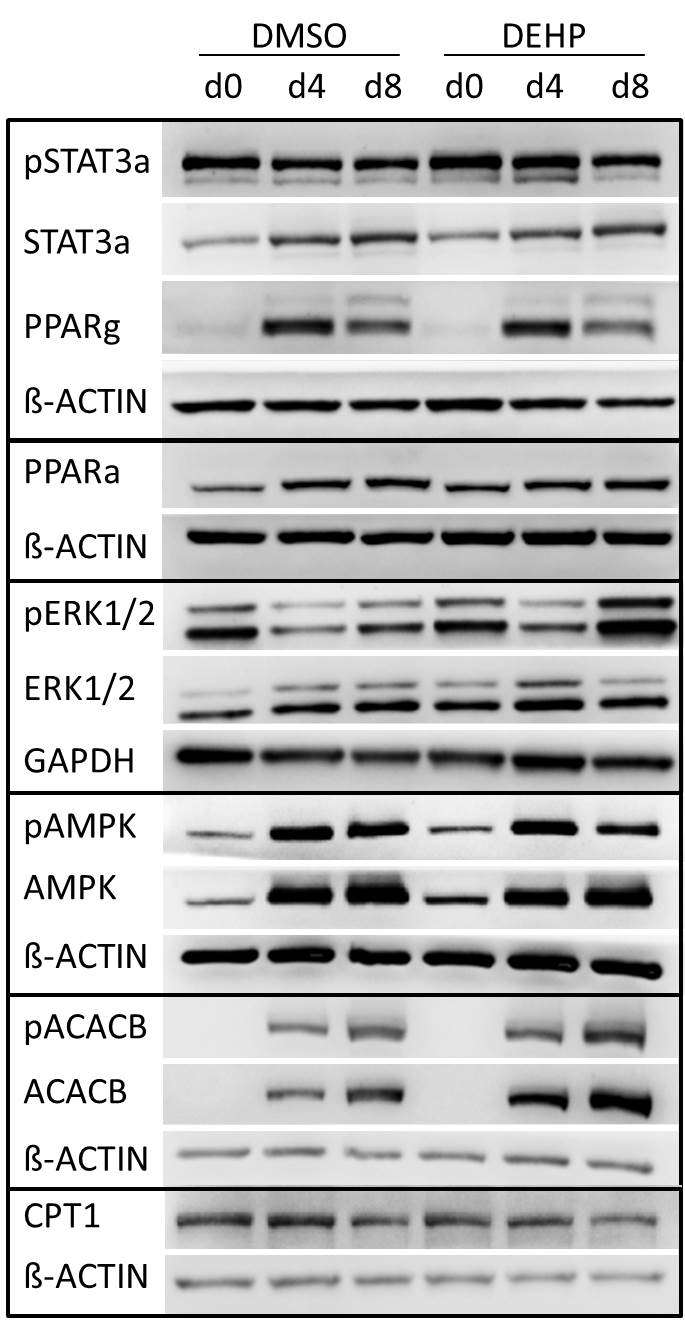


**Supplementary Figure S3: Western Blot images of analyzed proteins.** SGBS cells were exposed to DEHP from d0-d4 and subsequently differentiated into adipocytes. For western blot analysis samples were taken at d0, d4 and d8 of differentiation. After blotting, the membranes have been cut into slices to detect proteins of different sizes within one blot including the housekeeping proteins ß-actin or GAPDH. The different slices of a membrane are indicated by white space. All proteins that have been detected on slices of the same membrane are grouped and surrounded by the black frame.


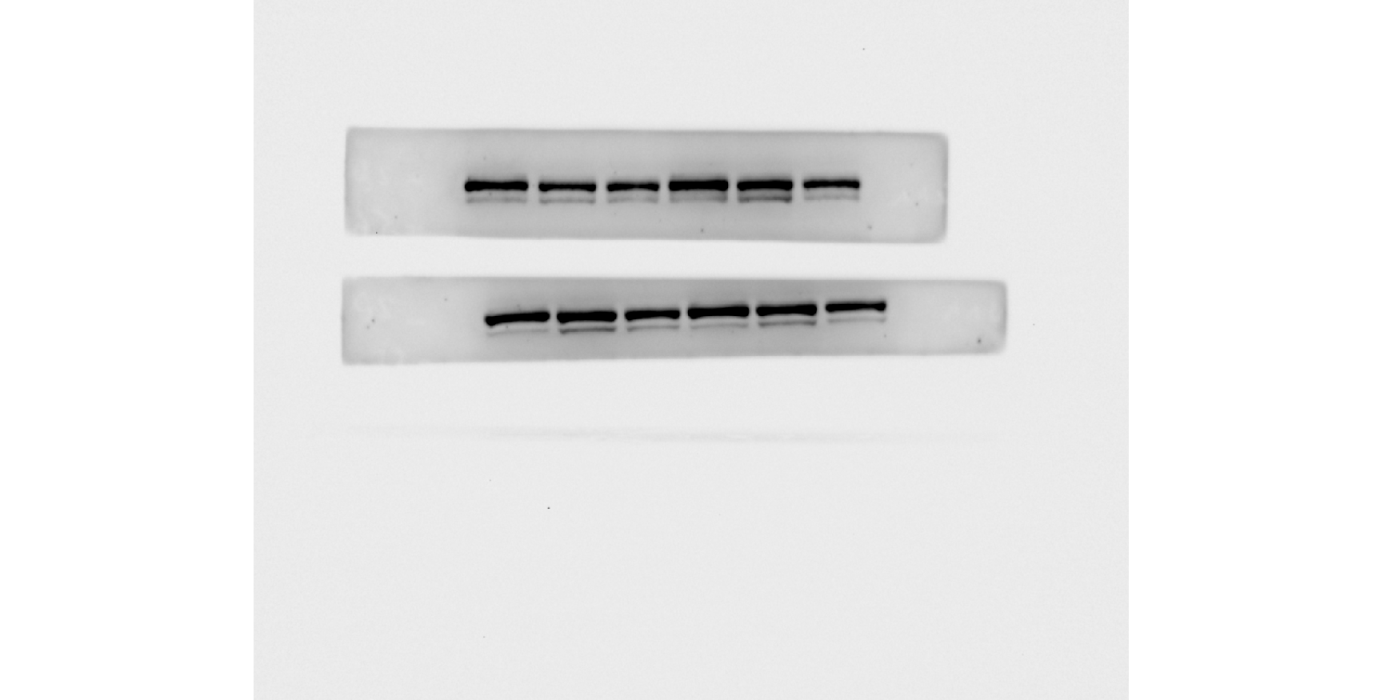


**Supplementary Figure S4: Western Blot image of pSTAT3a.** SGBS cells were exposed to DEHP from d0-d4 and subsequently differentiated into adipocytes. For western blot analysis samples were taken at d0, d4 and d8 of differentiation. After blotting, the membranes have been cut into slices to detect proteins of different sizes within one blot including the housekeeping proteins ß-actin or GAPDH. The image shows 2 slices, each from one independent experiment, which have been blotted in parallel (N=2 of N=4). The sample order is as followed: DMSO: d0, d4, d9; DEHP: d0, d4, d8. For the detection and quantification the Image Lab 5.2.1 software from BioRad has been used. Images that showed overexposure have been excluded from quantification.


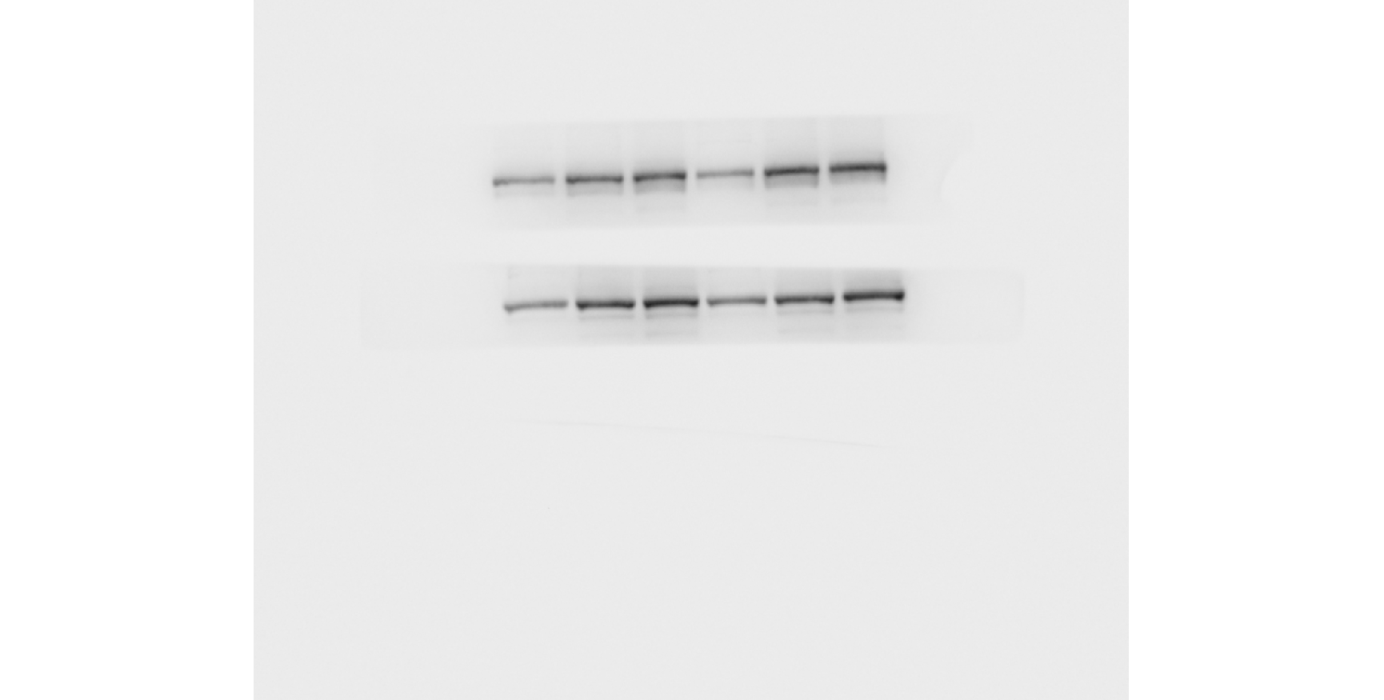


**Supplementary Figure S5: Western Blot image of STAT3a.** SGBS cells were exposed to DEHP from d0-d4 and subsequently differentiated into adipocytes. For western blot analysis samples were taken at d0, d4 and d8 of differentiation. After blotting, the membranes have been cut into slices to detect proteins of different sizes within one blot including the housekeeping proteins ß-actin or GAPDH. The image shows 2 slices, each from one independent experiment, which have been blotted in parallel (N=2 of N=4). The sample order is as followed: DMSO: d0, d4, d9; DEHP: d0, d4, d8. For the detection and quantification the Image Lab 5.2.1 software from BioRad has been used. Images that showed overexposure have been excluded from quantification.


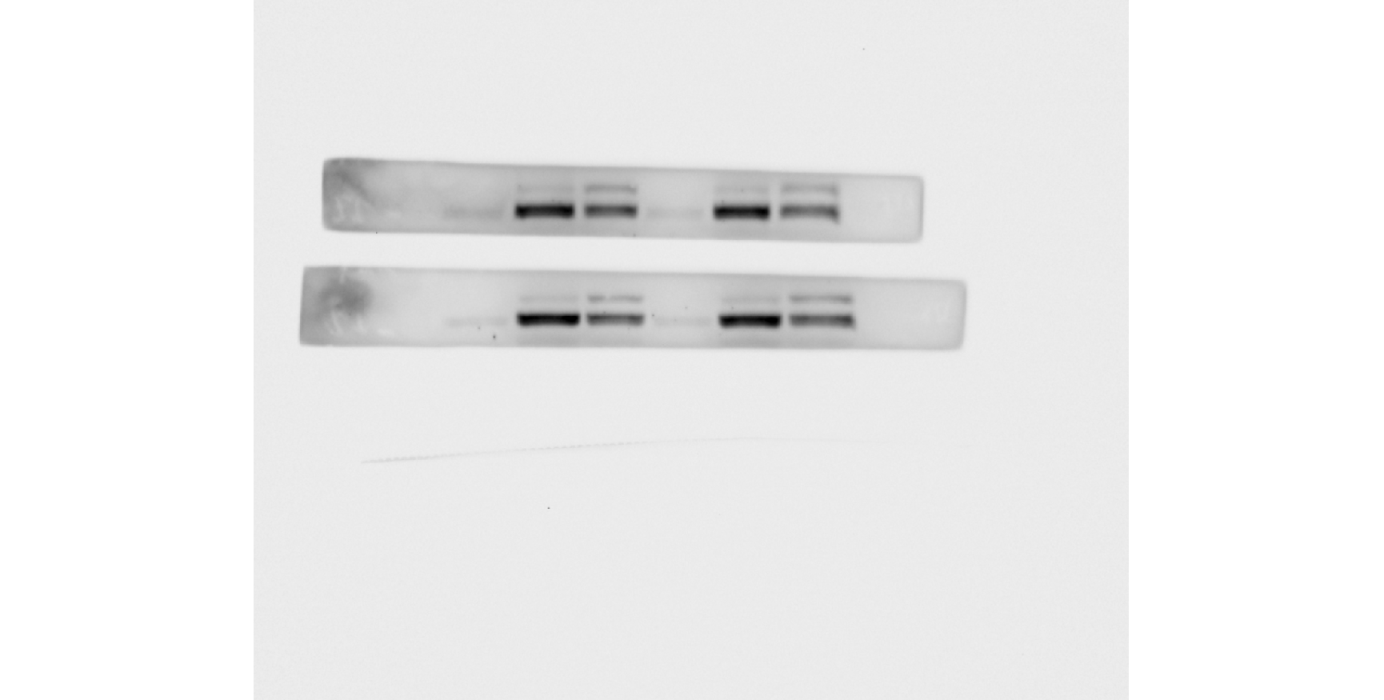


**Supplementary Figure S6: Western Blot image of PPARg.** SGBS cells were exposed to DEHP from d0-d4 and subsequently differentiated into adipocytes. For western blot analysis samples were taken at d0, d4 and d8 of differentiation. After blotting, the membranes have been cut into slices to detect proteins of different sizes within one blot including the housekeeping proteins ß-actin or GAPDH. The image shows 2 slices, each from one independent experiment, which have been blotted in parallel (N=2 of N=4). The sample order is as followed: DMSO: d0, d4, d9; DEHP: d0, d4, d8. For the detection and quantification the Image Lab 5.2.1 software from BioRad has been used. Images that showed overexposure have been excluded from quantification.


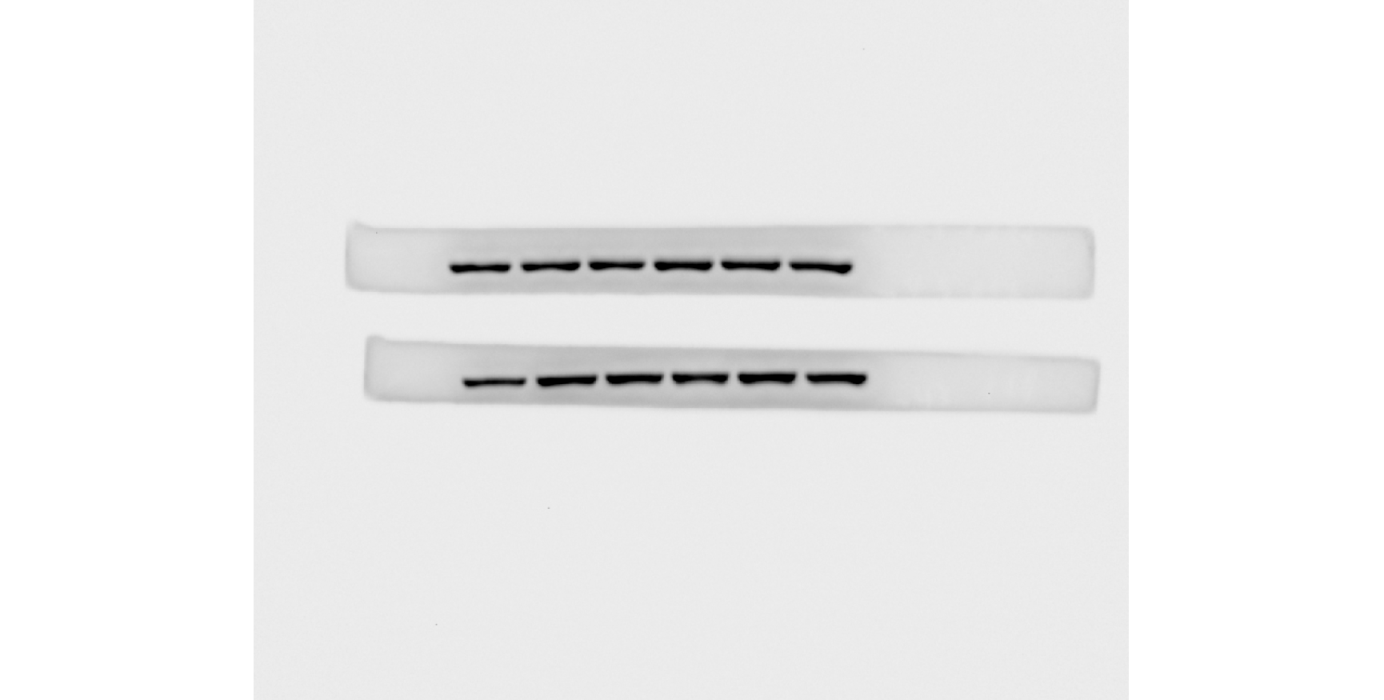


**Supplementary Figure S7: Western Blot image of ß-ACTIN for pSTAT3a, STAT3a and PPARg.** SGBS cells were exposed to DEHP from d0-d4 and subsequently differentiated into adipocytes. For western blot analysis samples were taken at d0, d4 and d8 of differentiation. After blotting, the membranes have been cut into slices to detect proteins of different sizes within one blot including the housekeeping proteins ß-actin or GAPDH. The image shows 2 slices, each from one independent experiment, which have been blotted in parallel (N=2 of N=4). The sample order is as followed: DMSO: d0, d4, d9; DEHP: d0, d4, d8. For the detection and quantification the Image Lab 5.2.1 software from BioRad has been used. Images that showed overexposure have been excluded from quantification.


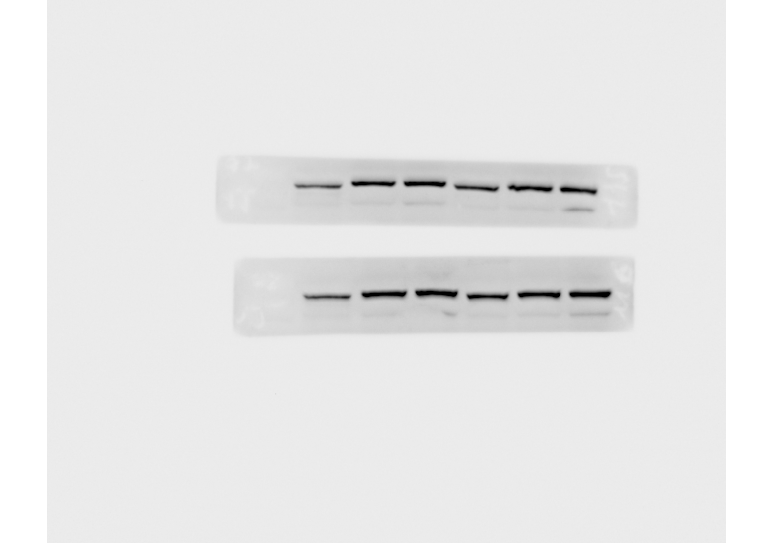


**Supplementary Figure S8: Western Blot image of PPARa.** SGBS cells were exposed to DEHP from d0-d4 and subsequently differentiated into adipocytes. For western blot analysis samples were taken at d0, d4 and d8 of differentiation. After blotting, the membranes have been cut into slices to detect proteins of different sizes within one blot including the housekeeping proteins ß-actin or GAPDH. The image shows 2 slices, each from one independent experiment, which have been blotted in parallel (N=2 of N=4). The sample order is as followed: DMSO: d0, d4, d9; DEHP: d0, d4, d8. For the detection and quantification the Image Lab 5.2.1 software from BioRad has been used. Images that showed overexposure have been excluded from quantification.


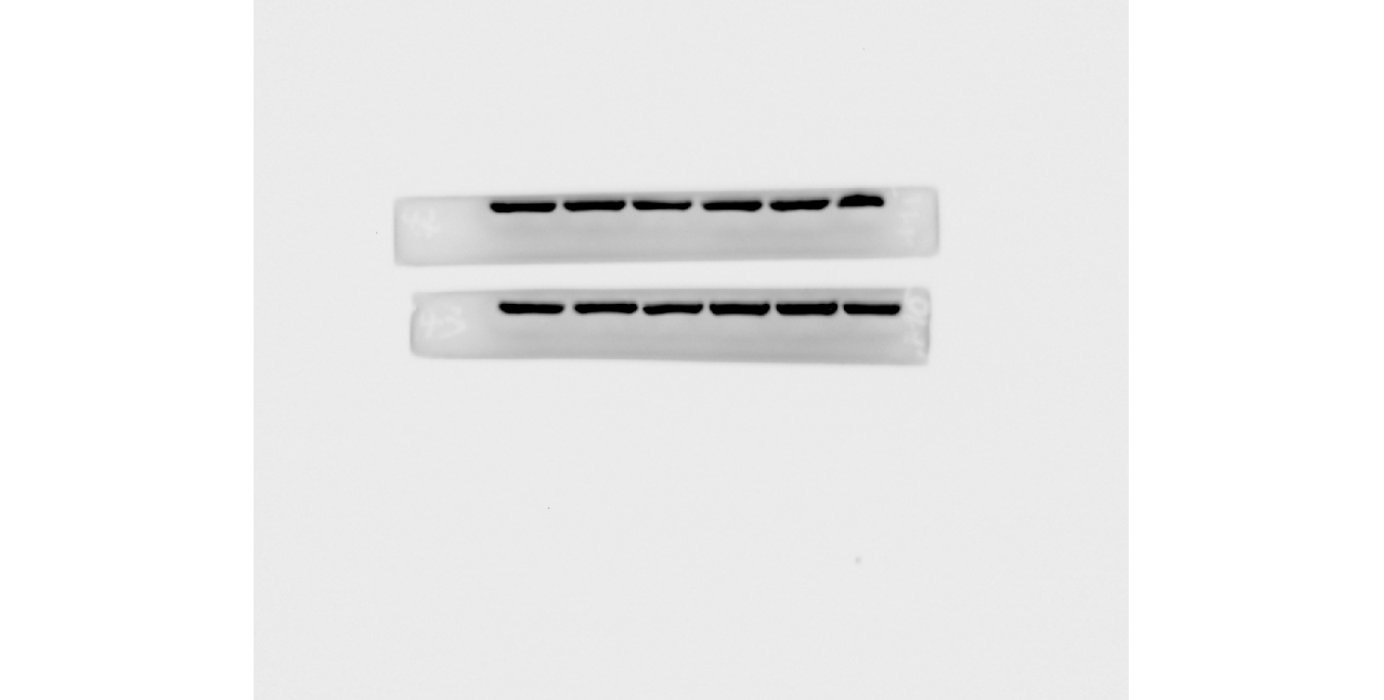


**Supplementary Figure S9: Western Blot image of ß-ACTIN for PPARa.** SGBS cells were exposed to DEHP from d0-d4 and subsequently differentiated into adipocytes. For western blot analysis samples were taken at d0, d4 and d8 of differentiation. After blotting, the membranes have been cut into slices to detect proteins of different sizes within one blot including the housekeeping proteins ß-actin or GAPDH. The image shows 2 slices, each from one independent experiment, which have been blotted in parallel (N=2 of N=4). The sample order is as followed: DMSO: d0, d4, d9; DEHP: d0, d4, d8. For the detection and quantification the Image Lab 5.2.1 software from BioRad has been used. Images that showed overexposure have been excluded from quantification.


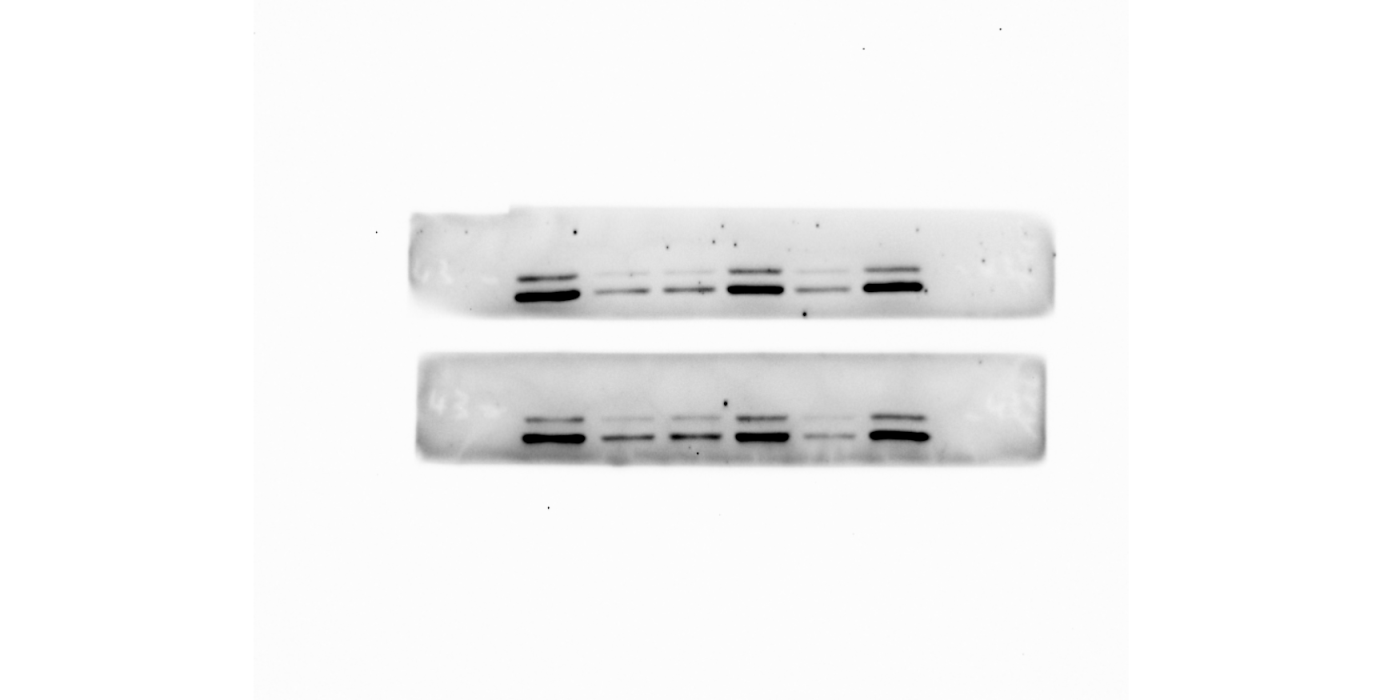


**Supplementary Figure S10: Western Blot image of pERK1/2.** SGBS cells were exposed to DEHP from d0-d4 and subsequently differentiated into adipocytes. For western blot analysis samples were taken at d0, d4 and d8 of differentiation. After blotting, the membranes have been cut into slices to detect proteins of different sizes within one blot including the housekeeping proteins ß-actin or GAPDH. The image shows 2 slices, each from one independent experiment, which have been blotted in parallel (N=2 of N=4). The sample order is as followed: DMSO: d0, d4, d9; DEHP: d0, d4, d8. For the detection and quantification the Image Lab 5.2.1 software from BioRad has been used. Images that showed overexposure have been excluded from quantification.


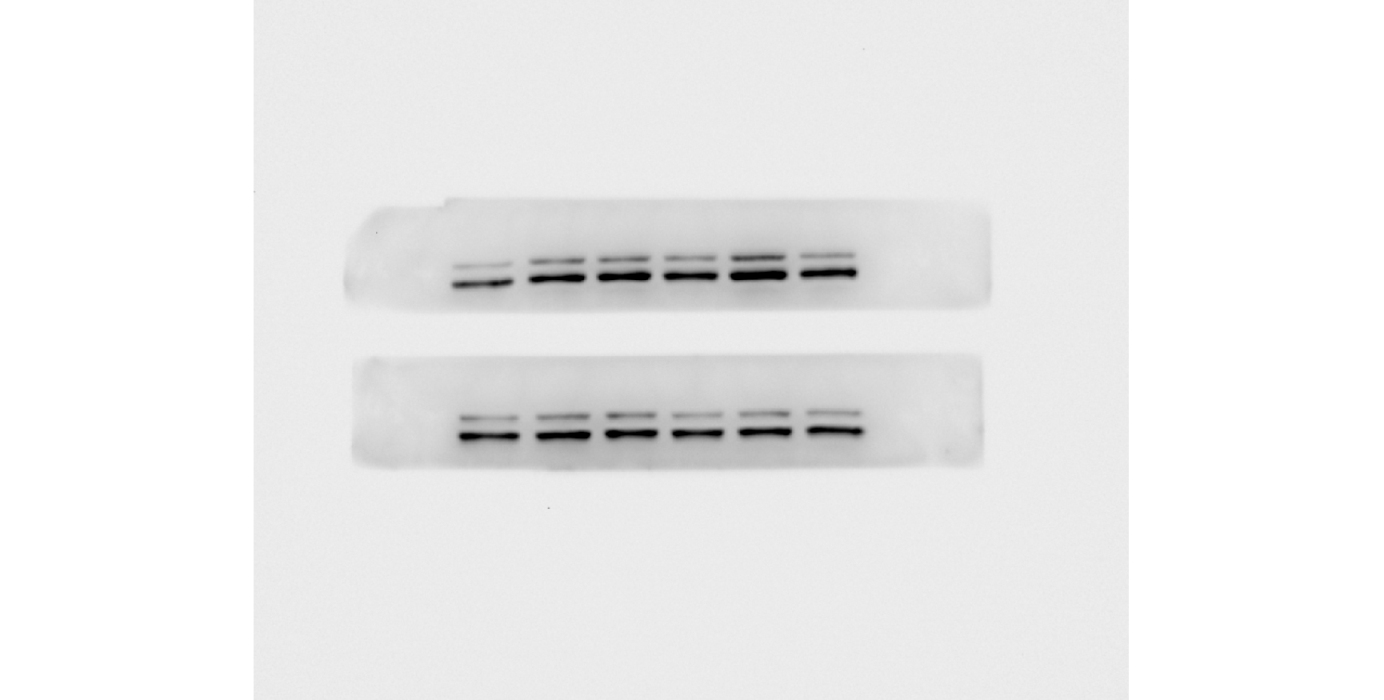


**Supplementary Figure S11: Western Blot image of ERK1/2.** SGBS cells were exposed to DEHP from d0-d4 and subsequently differentiated into adipocytes. For western blot analysis samples were taken at d0, d4 and d8 of differentiation. After blotting, the membranes have been cut into slices to detect proteins of different sizes within one blot including the housekeeping proteins ß-actin or GAPDH. The image shows 2 slices, each from one independent experiment, which have been blotted in parallel (N=2 of N=4). The sample order is as followed: DMSO: d0, d4, d9; DEHP: d0, d4, d8. For the detection and quantification the Image Lab 5.2.1 software from BioRad has been used. Images that showed overexposure have been excluded from quantification.


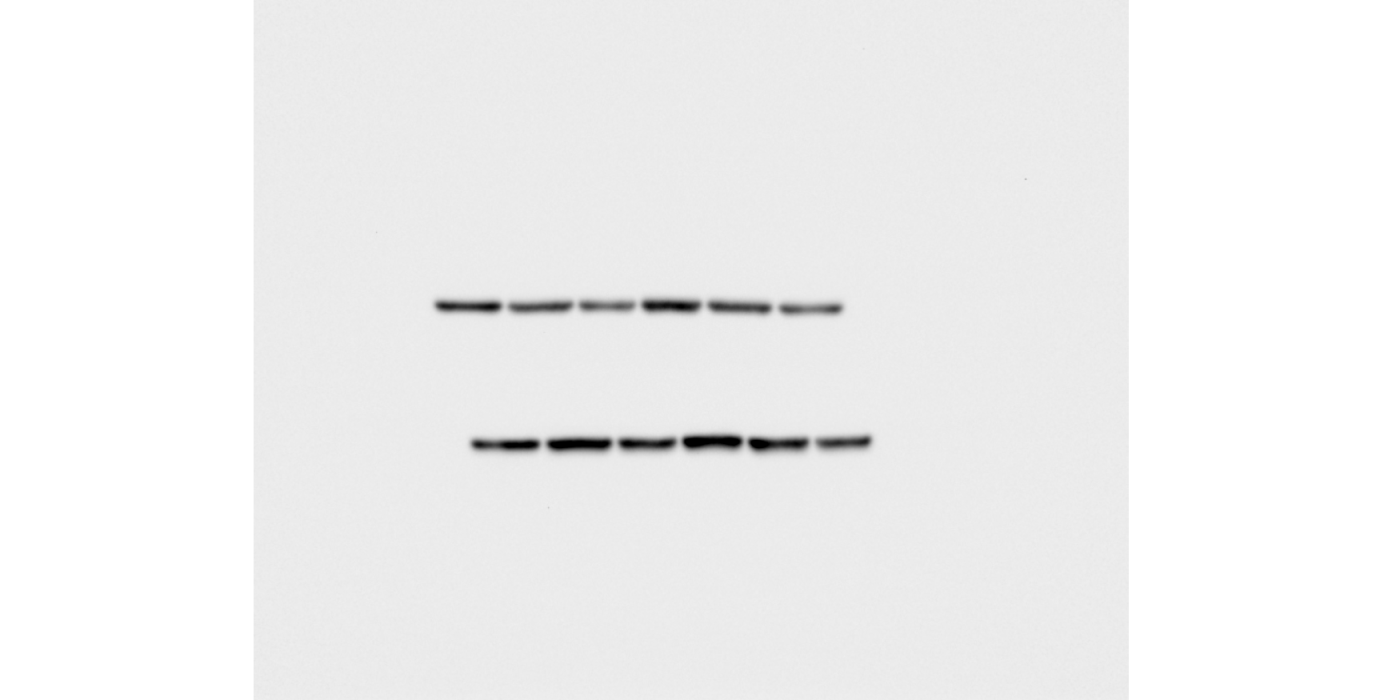


**Supplementary Figure S11: Western Blot image of GAPDH for pERK1/2 and ERK1/2.** SGBS cells were exposed to DEHP from d0-d4 and subsequently differentiated into adipocytes. For western blot analysis samples were taken at d0, d4 and d8 of differentiation. After blotting, the membranes have been cut into slices to detect proteins of different sizes within one blot including the housekeeping proteins ß-actin or GAPDH. The image shows 2 slices, each from one independent experiment, which have been blotted in parallel (N=2 of N=4). The sample order is as followed: DMSO: d0, d4, d9; DEHP: d0, d4, d8. For the detection and quantification the Image Lab 5.2.1 software from BioRad has been used. Images that showed overexposure have been excluded from quantification.


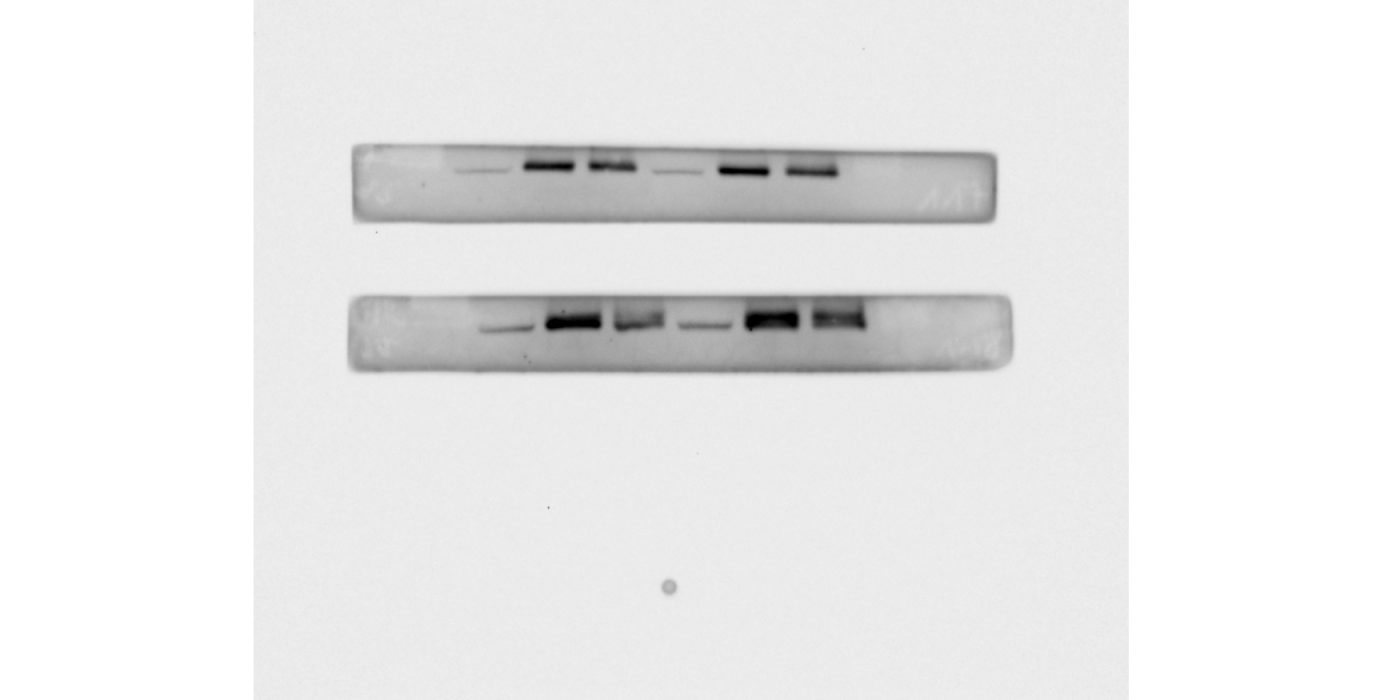


**Supplementary Figure S12: Western Blot image of pAMPK.** SGBS cells were exposed to DEHP from d0-d4 and subsequently differentiated into adipocytes. For western blot analysis samples were taken at d0, d4 and d8 of differentiation. After blotting, the membranes have been cut into slices to detect proteins of different sizes within one blot including the housekeeping proteins ß-actin or GAPDH. The image shows 2 slices, each from one independent experiment, which have been blotted in parallel (N=2 of N=4). The sample order is as followed: DMSO: d0, d4, d9; DEHP: d0, d4, d8. For the detection and quantification the Image Lab 5.2.1 software from BioRad has been used. Images that showed overexposure have been excluded from quantification.


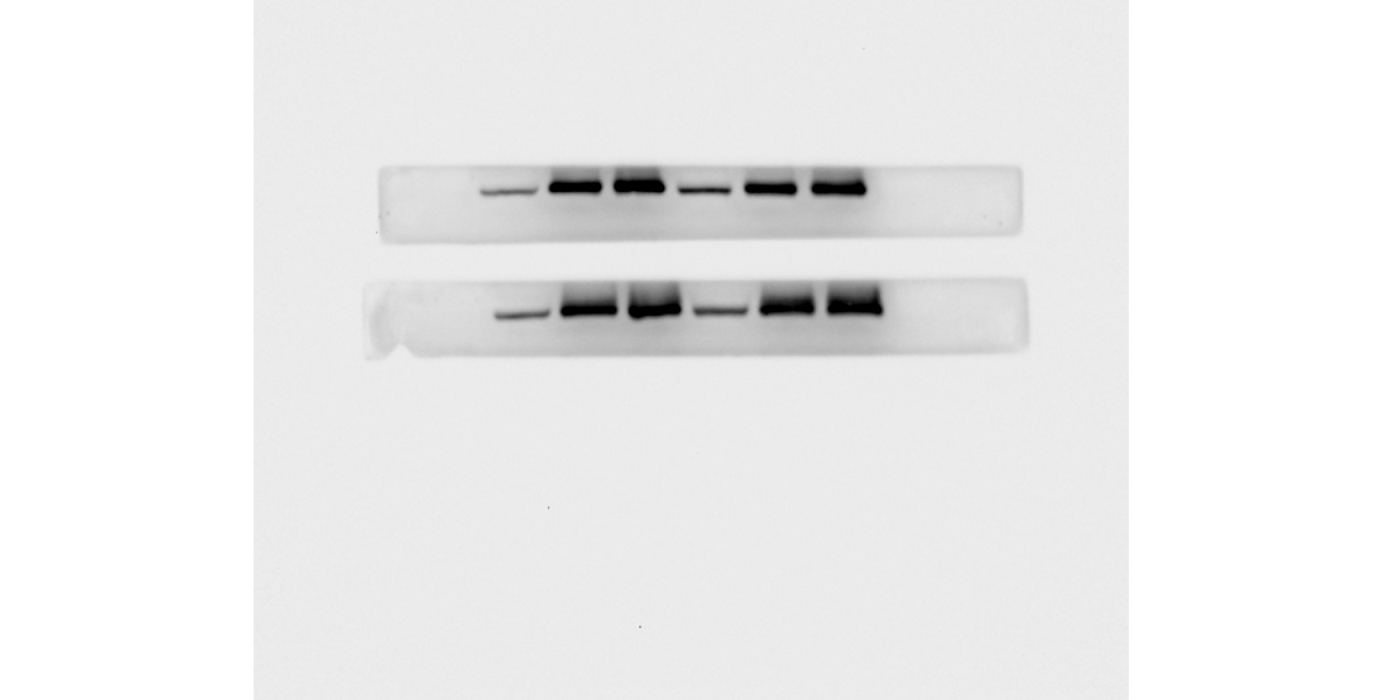


**Supplementary Figure S13: Western Blot image of AMPK.** SGBS cells were exposed to DEHP from d0-d4 and subsequently differentiated into adipocytes. For western blot analysis samples were taken at d0, d4 and d8 of differentiation. After blotting, the membranes have been cut into slices to detect proteins of different sizes within one blot including the housekeeping proteins ß-actin or GAPDH. The image shows 2 slices, each from one independent experiment, which have been blotted in parallel (N=2 of N=4). The sample order is as followed: DMSO: d0, d4, d9; DEHP: d0, d4, d8. For the detection and quantification the Image Lab 5.2.1 software from BioRad has been used. Images that showed overexposure have been excluded from quantification.


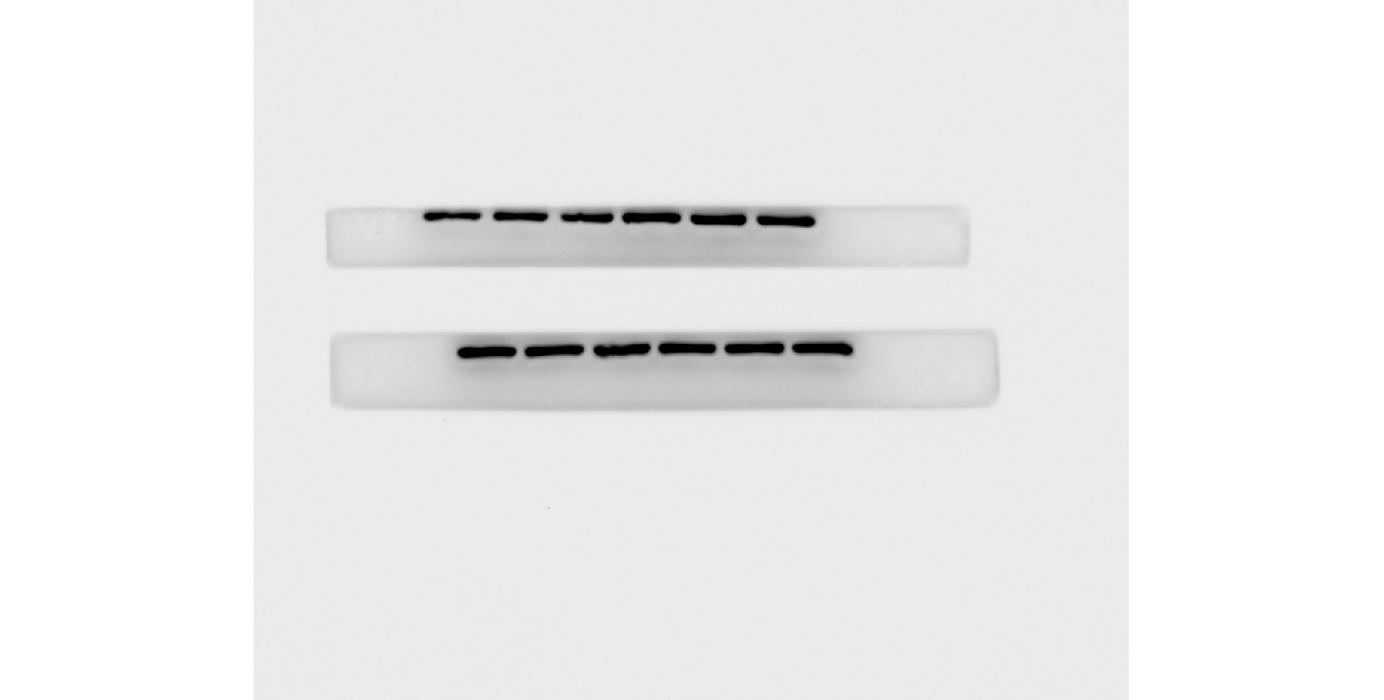


**Supplementary Figure S13: Western Blot image of ß-ACTIN for pAMPK and AMPK.** SGBS cells were exposed to DEHP from d0-d4 and subsequently differentiated into adipocytes. For western blot analysis samples were taken at d0, d4 and d8 of differentiation. After blotting, the membranes have been cut into slices to detect proteins of different sizes within one blot including the housekeeping proteins ß-actin or GAPDH. The image shows 2 slices, each from one independent experiment, which have been blotted in parallel (N=2 of N=4). The sample order is as followed: DMSO: d0, d4, d9; DEHP: d0, d4, d8. For the detection and quantification the Image Lab 5.2.1 software from BioRad has been used. Images that showed overexposure have been excluded from quantification.


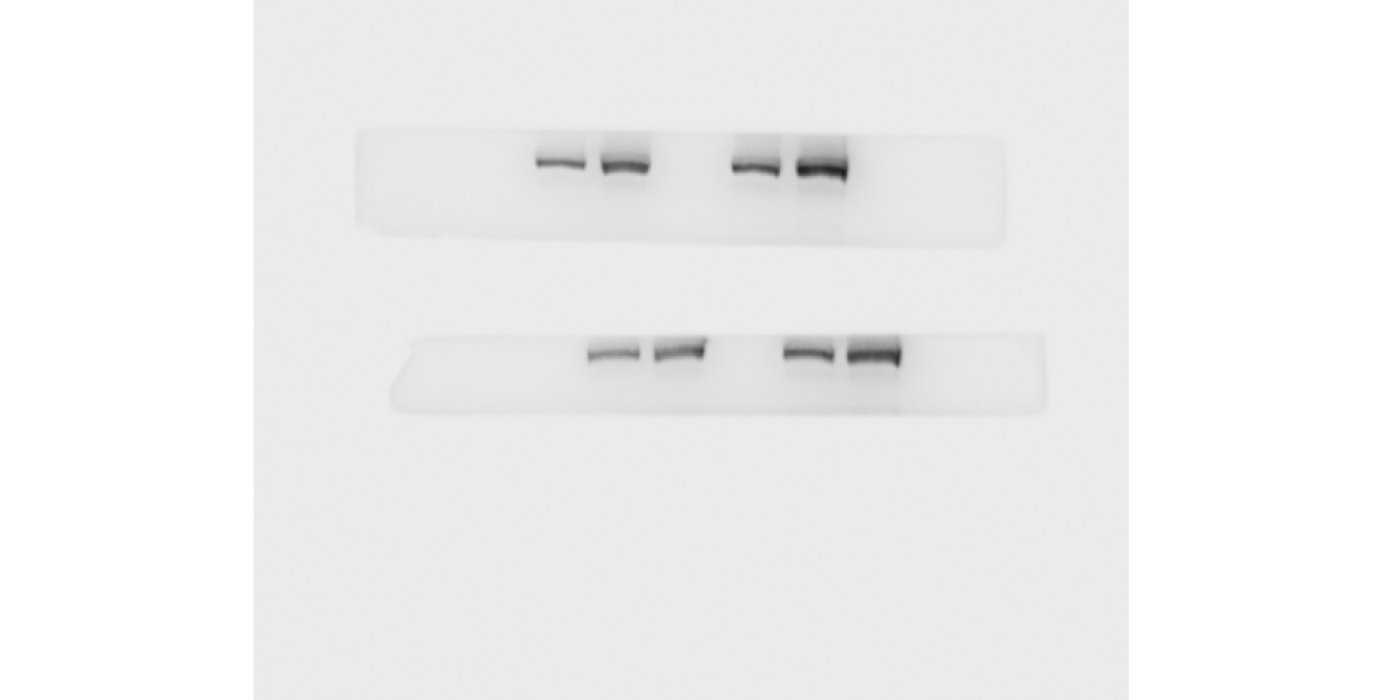


**Supplementary Figure S14: Western Blot image of pACACB.** SGBS cells were exposed to DEHP from d0-d4 and subsequently differentiated into adipocytes. For western blot analysis samples were taken at d0, d4 and d8 of differentiation. After blotting, the membranes have been cut into slices to detect proteins of different sizes within one blot including the housekeeping proteins ß-actin or GAPDH. The image shows 2 slices, each from one independent experiment, which have been blotted in parallel (N=2 of N=4). The sample order is as followed: DMSO: d0, d4, d9; DEHP: d0, d4, d8. For the detection and quantification the Image Lab 5.2.1 software from BioRad has been used. Images that showed overexposure have been excluded from quantification.


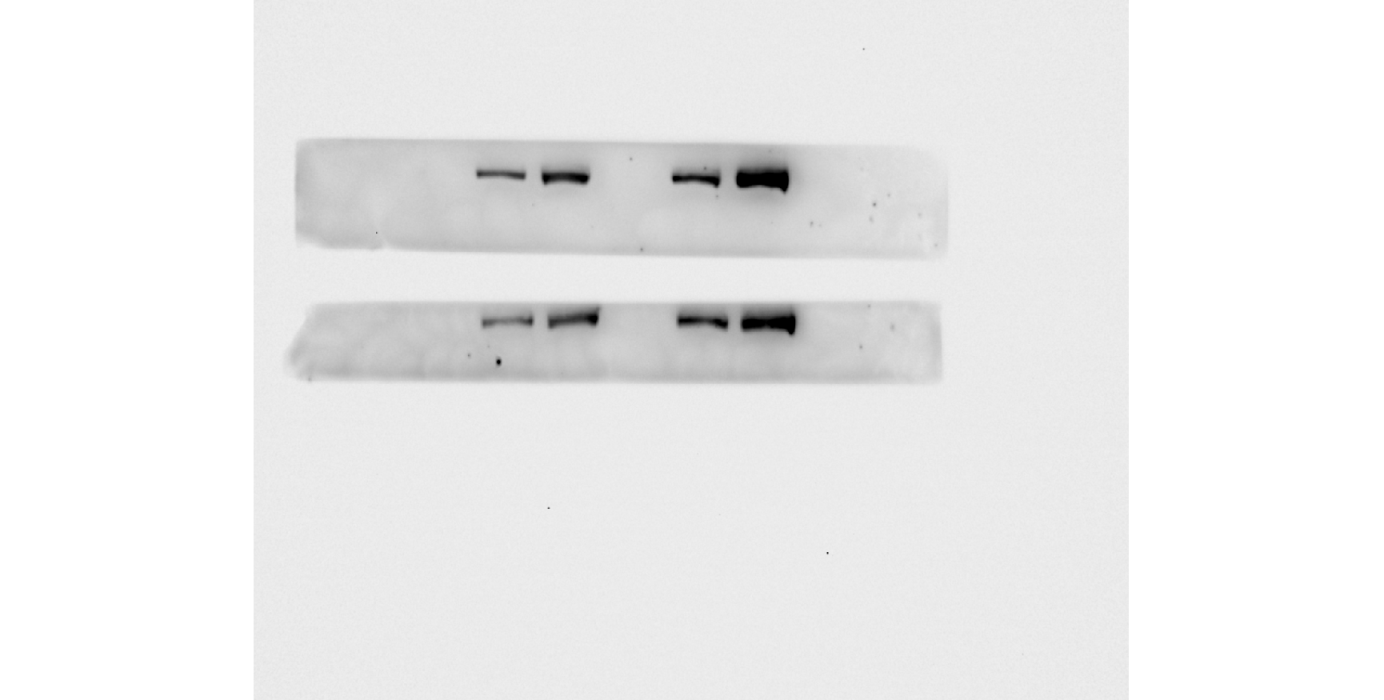


**Supplementary Figure S15: Western Blot image of ACACB.** SGBS cells were exposed to DEHP from d0-d4 and subsequently differentiated into adipocytes. For western blot analysis samples were taken at d0, d4 and d8 of differentiation. After blotting, the membranes have been cut into slices to detect proteins of different sizes within one blot including the housekeeping proteins ß-actin or GAPDH. The image shows 2 slices, each from one independent experiment, which have been blotted in parallel (N=2 of N=4). The sample order is as followed: DMSO: d0, d4, d9; DEHP: d0, d4, d8. For the detection and quantification the Image Lab 5.2.1 software from BioRad has been used. Images that showed overexposure have been excluded from quantification.


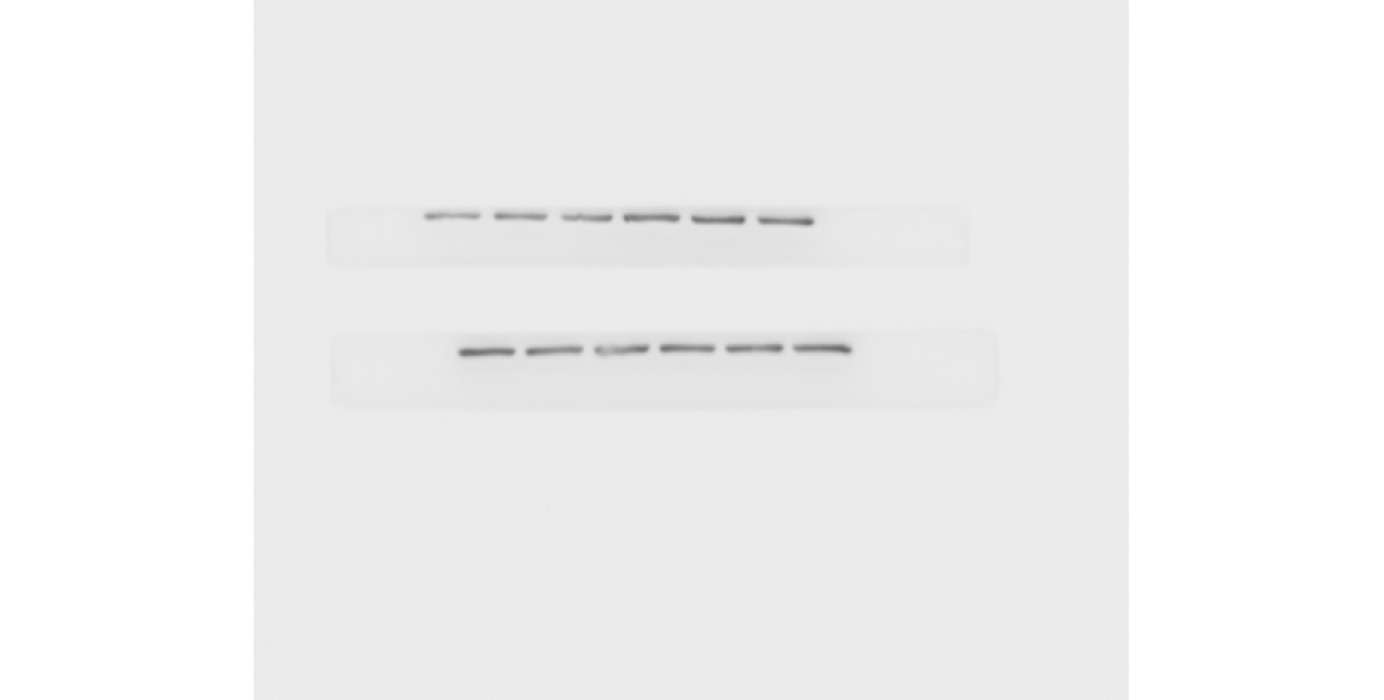


**Supplementary Figure S16: Western Blot image of ß-ACTIN for pACACB and ACACB.** SGBS cells were exposed to DEHP from d0-d4 and subsequently differentiated into adipocytes. For western blot analysis samples were taken at d0, d4 and d8 of differentiation. After blotting, the membranes have been cut into slices to detect proteins of different sizes within one blot including the housekeeping proteins ß-actin or GAPDH. The image shows 2 slices, each from one independent experiment, which have been blotted in parallel (N=2 of N=4). The sample order is as followed: DMSO: d0, d4, d9; DEHP: d0, d4, d8. For the detection and quantification the Image Lab 5.2.1 software from BioRad has been used. Images that showed overexposure have been excluded from quantification.
